# Insights into Effects/Risks of Chronic Hypergastrinemia and Lifelong PPI Treatment in Man Based on Studies of Patients with Zollinger–Ellison Syndrome

**DOI:** 10.3390/ijms20205128

**Published:** 2019-10-16

**Authors:** Lingaku Lee, Irene Ramos-Alvarez, Tetsuhide Ito, Robert T. Jensen

**Affiliations:** 1 Digestive Diseases Branch, NIDDK, NIH, Bethesda, MD 20892-1804, USA; frkn505@gmail.com (L.L.); irene.ramosalvarez@nih.gov (I.R.-A.); 2 Department of Medicine and Bioregulatory Science, Graduate School of Medical Sciences, Kyushu University, 3-1-1 Maidashi, Higashi-Ku, Fukuoka 812-8582, Japan; 3 Neuroendocrine Tumor Centra, Fukuoka Sanno Hospital, International University of Health and Welfare 3-6-45 Momochihama, Sawara-Ku, Fukuoka 814-0001, Japan; itopapa@kouhoukai.or.jp

**Keywords:** gastrinoma, hypergastrinemia, gastric carcinoid, PPI, neuroendocrine tumor, MEN1

## Abstract

The use of proton pump inhibitors (PPIs) over the last 30 years has rapidly increased both in the United States and worldwide. PPIs are not only very widely used both for approved indications (peptic ulcer disease, gastroesophageal reflux disease (GERD), Helicobacter pylori eradication regimens, stress ulcer prevention), but are also one of the most frequently off-label used drugs (25–70% of total). An increasing number of patients with moderate to advanced gastroesophageal reflux disease are remaining on PPI indefinitely. Whereas numerous studies show PPIs remain effective and safe, most of these studies are <5 years of duration and little data exist for >10 years of treatment. Recently, based primarily on observational/epidemiological studies, there have been an increasing number of reports raising issues about safety and side-effects with very long-term chronic treatment. Some of these safety issues are related to the possible long-term effects of chronic hypergastrinemia, which occurs in all patients taking chronic PPIs, others are related to the hypo-/achlorhydria that frequently occurs with chronic PPI treatment, and in others the mechanisms are unclear. These issues have raised considerable controversy in large part because of lack of long-term PPI treatment data (>10–20 years). Zollinger–Ellison syndrome (ZES) is caused by ectopic secretion of gastrin from a neuroendocrine tumor resulting in severe acid hypersecretion requiring life-long antisecretory treatment with PPIs, which are the drugs of choice. Because in <30% of patients with ZES, a long-term cure is not possible, these patients have life-long hypergastrinemia and require life-long treatment with PPIs. Therefore, ZES patients have been proposed as a good model of the long-term effects of hypergastrinemia in man as well as the effects/side-effects of very long-term PPI treatment. In this article, the insights from studies on ZES into these controversial issues with pertinence to chronic PPI use in non-ZES patients is reviewed, primarily concentrating on data from the prospective long-term studies of ZES patients at NIH.

## 1. Introduction

Proton pump inhibitors (PPIs) inhibit gastric H^+^K^+^ATPase, which is required for gastric acid secretion, and are one of the most widely used classes of drugs in the world [1,2]. PPIs are not only very widely used both for approved indications (peptic ulcer disease, gastroesophageal reflux disease (GERD), Helicobacter pylori eradication regimens, stress ulcer prevention), but are also one of the most frequently off-label used drugs (25–70% of total) [3], with the result that their use over the last decade has increased by >3 times in many countries and they are in the top 10 drugs prescribed [4,5,6]. PPIs have been increasingly used for >30 years, have proven effective, and in most reviews are considered safe [1,2,7,8,9]. However, there are currently a number of contentious issues related to their safety [2,3,7,10,11,12,13,14,15,16,17], primarily based on recent observational/epidemiological studies. At present, there are increasing reports of potential significant side-effects with long-term PPI use, which is leading to increasing debate on their safety, especially with very long/lifetime use [2,3,7,13,14,15,16,18,19]. These safety issues are in large part thought to be due to chronic hypergastrinemia and hypochlorhydia/achlorhydria that develop with long-term PPI treatment. Despite PPIs extended use in many patients, the risk of very long-term/lifetime treatment in humans is still unclear. 

Zollinger–Ellison syndrome (ZES), which is due to ectopic secretion of gastrin from a neuroendocrine tumor (usually in the pancreas/duodenum) resulting in severe, recalcitrant peptic ulcer disease/gastro-esophageal reflux disease (GERD) [20,21,22] has been proposed to be a good model to study the lifelong effects of chronic hypergastrinemia in man [19,21,23,24,25,26,27]. Furthermore, the majority of ZES patients require lifelong treatment for the marked gastric acid hypersecretion they develop, with potent gastric acid anti-secretory agents, for which PPIs are now the drugs of choice [19,22,28,29,30,31]. ZES patients were one of the earliest groups of patients treated with PPIs [32,33,34] and PPI use in these patients was one of their first worldwide approved indications for their use; therefore, many ZES patients have been taking PPIs continuously, often with high doses [27,30,31,34,35,36], for >30 years. Therefore, ZES patients are an excellent natural model to study to provide potential insights into the risks of lifelong hypergastrinemia, as well as in the possible effects of lifelong PPI use.

In this paper, each of these two areas will be reviewed from studies in ZES patients to provide insights into the increasing debate on the long-term safety of PPIs and risks of very long-term chronic hypergastrinemia. Before this is addressed, it is important to understand why chronic hypergastrinemia is considered a health concern, what are the specific safety concerns being raised currently with PPIs, and how the study of ZES is well-versed to address some of these issues.

## 2. Chronic Hypergastrinemia: General

Chronic hypergastrinemia, which has both long-fascinated investigators/clinicians, as well as being a constant source of contention and puzzlement, has recently been receiving increased attention [10,37,38,39,40,41]. It has long fascinated investigators/clinicians because of its multiple causes including diseases such as pancreatic neuroendocrine tumors (panNET) and other neuroendocrine tumors (NETs) causing fasting gastrin levels >100-fold increased, associated with such florid acid hypersecretion that if untreated (i.e., ZES), it frequently leads to a fatal outcome [24,28,29,42]. It is contentious and puzzling, because the diagnosis can be difficult to establish, and the very long-term/lifelong effects in humans of chronic hypergastrinemia due to agents such as PPIs is still relatively unknown. This has led to the result that the very long-term potential consequences of chronic hypergastrinemia in humans have been much debated, in large part due to the diverse reported effects of gastrin in both normal and pathological processes, especially from animal studies/in vitro systems/experimental systems [10,12,18,38,43,44]. Furthermore, in animal models, chronic hypergastrinemia has been shown to stimulate the proliferation of gastric enterochromaffin-like cells (ECL cells) and the development of gastric carcinoid tumors [19,45,46,47,48,49]. Chronic hypergastrinemia is currently receiving increased attention, primarily because of it increased occurrence due to the increased worldwide long-term use of potent gastric acid anti-suppressant drugs, such as proton pump inhibitors (PPIs) (gastric H^+^K^+^ATPase inhibitors) (omeprazole, lansoprazole, dexlansoprazole, esomeprazole, pantoprazole, rabeprazole) [4,5,6,17]. Each of these points will be discussed in more detail in the following paragraphs.

Since the development of sensitive gastrin radio-immunoassays in the 1960s, it has become clear that chronic hypergastrinemia is not an infrequent finding and that it has both physiologic and pathophysiological causes [10,37,38,39,40,41]. The physiological causes can be a response to anything that results in prolonged hypo/achlorhydria, which can include processes as varied as the chronic use of potent acid suppressive drugs, gastric infections such as Helicobacter pylori, and postsurgical procedures such as vagotomy or other gastric acid-reducing surgeries [10,37,38,39,40,41]. Pathological causes of chronic hypergastrinemia include any disease causing hypo-/achlorhydria (including potent gastric anti-secretory drugs such as PPIs, pernicious anemia, atrophic gastritis, failure to develop functional parietal cells because of an inherited disorder such as defects in the genes encoding the gastric H^+^K^+^ acid pump) [10,39,40,41,50]; overproduction of gastrin due to the presence of NETs ectopically secreting gastrin (gastrinoma), as well as other disease states such as chronic gastric infections such as Helicobacter pylori, renal failure, and massive small bowel resections [10,21,37,38,39,40,41].

## 3. Why is Chronic Hypergastrinemia from PPI Use Receiving Increased Attention and Generating Continued Debate?

PPIs inactivate the gastric H^+^K^+^ATPase responsible for generating gastric acid and have very long durations of action [1,2,33,38,51,52]. In contrast, the other class of drugs widely used as gastric acid suppressants, the histamine H_2_-receptor antagonists (cimetidine, ranitidine, famotidine, etc.), all have a relatively short durations of action for inhibiting acid secretion, that extends less than 6–15 h after stopping the drug, whereas the actions of PPIs can last up to a week [1,2,17,33,51,52,53]. This prolonged action of PPIs is due to the fact that the generation of new, active gastric H^+^K^+^ATPase is required to recover acid secretion after PPI use [1,2]. The result of this profound inhibition of acid secretion is that PPIs are highly effective for the treatment of gastric esophageal reflux disease (GERD), which requires marked acid inhibition to control symptoms in many patients [1,2,7]. A consequence of this marked acid inhibition is the stimulation of the physiological response to hypo-/achlorhydria, which involves stimulation of G cells in the gastric antrum to release gastrin and hypergastrinemia results if acid secretion does not recur to sufficient levels to counteract this reflex. The generation of hypergastrinemia occurs with PPI use rapidly and can be seen in 80–100% of patients taking long-term PPIs [17,18,38,54]. In most cases, the fasting serum gastrin (FSG) levels increase <3–4-fold above normal, but in a proportion of patients (10–30%), the FSG levels increases >5-fold elevated, which is in the range of what is frequently seen in ZES patients [17,18,24,54,55,56]. Because GERD symptoms rapidly return when PPIs are stopped in non-ZES patients with moderate–severe GERD, many patients continue taking PPIs long-term, and in addition, because a proportion began taking PPIs at younger ages, very long-term PPI treatment for years is increasingly occurring [1,2]. 

Whereas many studies show long-term PPI treatment is well-tolerated, tolerance to the PPIs does not occur and PPIs remain effective [1,2,7]. The major factor leading to debate is the question of the safety of very long-term/lifetime treatment. Initial toxicity studies with omeprazole showed that long-term treatment of rats, but not mice, led to the development of gastric carcinoid tumors [45,46]. Subsequent animal studies using different methods to induce hypergastrinemia (other gastric anti-secretory drugs, gastrin infusions, surgical procedures (partial fundectomy)) demonstrated, they each resulted in proliferation of gastric enterochromaffin-like cells (ECL cells), and in some cases (rats, mastomys), to the development of carcinoid tumors, which on occasion were malignant [25,44,45,46,48,57]. Gastric carcinoid tumors are now classified into three subtypes by some [58,59] and into four subtypes by others [60], including: Type 1 gastric carcinoids, which are sporadic in nature, gastrin-dependent, well-differentiated, arise in chronic hypergastrinemic states such as chronic atrophic gastritis/pernicious anemia, comprise 70–80% of all gastric carcinoids, and metastasize in <10% [58,59,60]. Type 2 gastric carcinoids occur in ZES patients, primarily in patients with multiple endocrine neoplasia type 1(MEN1/ZES), comprise 5–6% of all gastric carcinoids, are well-differentiated, are also gastrin-dependent, and malignant in 10–30% [58,59,60]. Type 3 gastric carcinoids are gastrin-independent, sporadic in occurrence, are well-differentiated, comprise 14–25% of all gastric carcinoids, and are malignant in 25–40% [58,59,60]. Type 4 gastric carcinoids are sporadic in nature, gastrin-independent, poorly differentiated neuroendocrine tumors, comprise 6–8% of all gastric carcinoids, and metastasize in 50–100% [58,59,60]. It is proposed that Type 1 and 2 gastric carcinoid tumors originate from ECL cells with proliferation stimulated primarily by hypergastrinemia, and that they develop through a progression involving various ECL cell hyperplastic stages to dysplasia and carcinoid formation [43,47]. The continued question of long-term safety of PPIs on this issue occurs because it remains unclear to many what degrees of advanced proliferative effects and what frequency of Type 1 or 2 gastric carcinoids there will be seen in human gastric ECL cells during very long-term/lifetime PPI treatment.

What is clear at present is that in humans, chronic hypergastrinemic conditions (chronic PPI treatment, pernicious anemia, atrophic gastritis, Zollinger–Ellison syndrome, inherited defects of the proton pump, etc.) are associated with proliferative changes in gastric ECL cells [17,25,36,43,44,47,56,61,62]. Furthermore, a number of these chronic hypergastrinemic conditions such as pernicious anemia, atrophic gastritis, and lack of gastric ATPase genes are associated with the development of gastric carcinoid tumors [10,11,25,44,60,62,63]. Moreover, recent studies in humans support the role of chronic hypergastrinemia in inducing these ECL cell changes, as well as gastric carcinoids in the disease states above, with treatment with the CCK2-R (gastrin-receptor) antagonist, netazapide, demonstrating that its long-term use reverses these findings [64,65]. However, even with prolonged use of PPIs in man, there are only very uncommon reports of an association of PPI use with the development of gastric carcinoid tumors [66,67,68,69,70,71,72], and the evidence for such an association in some of the reports is too limited to firmly establish the association [2]. In studies in chronic atrophic gastritis, which is the most frequent chronic hypergastrinemia state studied, the chronic hypergastrinemia is accompanied by gastritis, mucosal atrophy, or both, and the presence of both correlates directly with the severity of the ECL cell changes [25,73,74]. Therefore, in this condition, it is not possible to isolate the effect of the chronic hypergastrinemia alone on the ECL cell proliferative changes [25,47]. In patients with chronic atrophic gastritis, the development with chronic hypergastrinemia of advanced ECL changes and carcinoid formation also correlated with the magnitude of the hypergastrinemia, gender, older age, the presence of intestinal metaplasia in the gastric mucosa, and higher serum chromogranin A (CgA) levels [25,75,76]. The low occurrence of gastric carcinoids in patients with chronic hypergastrinemia without chronic atrophic gastritis, inflammation, atrophy, or additional genetic factors has led to the proposal that in man chronic hypergastrinemia alone does not lead to progressive changes beyond ECL cell hyperplasia [2,73,77].

In addition to the well-established effect of gastrin in stimulating proliferation of gastric ECL cells, numerous studies in animals as well as in vitro studies have reported evidence that gastrin or gastrin precursors have stimulatory effects on the growth/development of a number of other neoplasms. These include particularly gastric cancer, esophageal cancers, pancreatic cancer, and colon cancers [2,78,79,80,81,82,83,84,85,86,87]. At present, the evidence that a neoplasm-associated growth affect is due to the hypergastrinemia or the exact role of gastrin in the growth or pathogenesis of these tumors in human chronic hypergastrinemia disorders is not compelling and is a source of debate. 

The above results, with a rapidly increasing and prolonged use of PPIs coupled with their ability to cause chronic hypergastrinemia with proliferative gastric ECL cell changes in man; their ability to cause gastric carcinoid tumors in rodents, but not humans with chronic PPI use over <5 years; and the experimental result of gastrin’s effect on a number of other cancer’s growth have led to debate over their safety during prolonged, life-time usage. 

## 4. Why is Prolonged PPI Use Receiving Increased Attention and Generating Continued Debate?

In addition to the question of the long-term possibility of chronic hypergastrinemia-induced gastric carcinoids, in a number of other recent specific studies, as well as observational and epidemiological studies, other possible PPI-induced side-effects with potential long-term safety considerations are being reported, which are attributed to the PPI-induced chronic hypergastrinemia, hypoachlorhydria, to other mechanisms, or due to unknown mechanisms [2,3,7,8,9,11,13,14,15,16,38,44]. 

Also receiving increased attention are the possible effects of chronic hypergastrinemia on non ECL cell tumor growth, as well as a number of primarily observational/epidemiological recent studies reporting an association of chronic PPI use with the development renal diseases; increasing the incidence of various lung and GI infections; affecting the absorption of various nutrients; participating in drug interactions that have therapeutic implication; contributing to bone fractures; and contributing to the development of important CNS diseases such as dementia [1,2,3,7,8,13,14,16,18,88]. 

All of the above reported side-effects are increasingly attributed to chronic PPI use and are a source of debate. With existing information, it is even more uncertain what may be their associations with very long/lifelong PPI treatment. In general, in most expert opinion/expert reviews, the current opinion is that PPIs are safe and effective over the <5-year follow-up periods analyzed. This conclusion was recently supported by results from a large, multicenter study [8] involving 17,598 participants in a randomized placebo-controlled study in which patients did or did not receive the PPI, pantoprazole, for a median of 3.01 years. This study [8] reported that the only possible PPI-induced safety difference detected [8] was an increased incidence of enteric infections in the PPI-treated patients. In an accompanying editorial [9], it was concluded from a review of this study that PPI treatment for appropriate indications can be safely continued for a few years with no increased risk for several reported potential side-effects except for a risk of gastrointestinal infections. 

## 5. Why Would Results from the Long-Term Study of Zollinger–Ellison Syndrome (ZES) Provide Useful Insights into the Issue of Safety of Chronic Hypergastrinemia from Lifelong Use of PPIs in nonZES Patients?

There are a number of reasons that ZES is an excellent model to study the effects of chronic, lifelong hypergastrinemia in man, with >99% of patients have fasting hypergastrinemia due to the ectopic secretion of gastrin from a neuroendocrine tumor (gastrinoma) [22,24].

**Figure 1:**
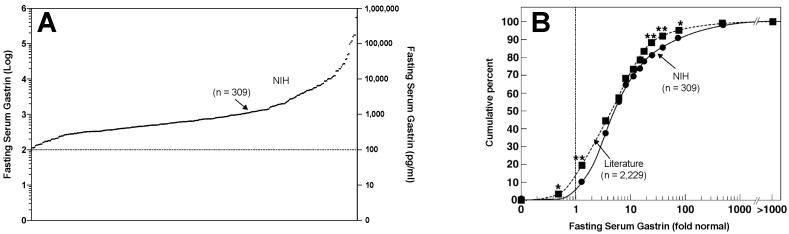
Fasting serum gastrin (FSG) concentration in Zollinger–Ellison syndrome (ZES) patients at NIH (*n* = 309) (**A**,**B**) and from the literature (*n* = 2229) (**B**). In (**A**), the FSG is expressed as log of concentration (left Y axis) with the numerical value in pg/mL (right Y axis), with upper limit of normal shown by the dotted line. In (**B**), the FSG levels from both the NIH and from literature patients are shown as fold over normal with normal FSG level indicated by the dotted line. Asterisks indicated statistically significant differences (*p* < 0.02) in two groups of patients for a given FSG level in (**B**). Figure is drawn from data in [24]. Note that 40% of ZES patients have FSG levels that overlap with those seen in non-ZES patients taking chronic proton pump inhibitors (PPIs).

First, the mean level of the hypergastrinemia in ZES patients is 4- to 5-fold elevated with 30–45% of the patients having FSG levels in the range seen with patients chronically taking PPIs (<4-fold elevated), and 35% of the patients having very high levels 10–100-fold elevated [22,24] (Figure 1). Furthermore, they have high levels of amidated gastrin’s, which are the form that causes acid secretion and ECL cell changes [24,39], as well as incompletely processed gastrin forms progastrin, and glycine-extended precursors, which are the forms that are reported to have growth affects in the colon, a number of other tissues and in other various tumors including colon cancer, although the latter points are controversial [21,24,39,78,89,90,91]. 

Secondly, the hypergastrinemia is lifelong in most patients (Table 1). This occurs because <30% of patients are cured lifelong [23,51,92,93,94,95], even though numerous detailed tumor localization methods are performed including cross-sectional imaging, hormonal gradient sampling, somatostatin receptor imaging, endoscopic ultrasound examinations [92,96,97,98,99,100,101,102,103,104], as well as specific intraoperative tumor localization methods such as performing a duodenotomy, transillumination of duodenum at surgery, extended Kocher maneuvers, and intraoperative ultrasound studies [92,94,105,106,107]. Furthermore, higher cure rates are not seen because 70–90% of patients have duodenal gastrinomas, which can be small (<0.5 cm), multiple, associated with positive lymph nodes, and easily missed at surgery [94,108,109,110,111,112]. Furthermore, up to 30% of patients present with liver metastases that are not completely resectable [21,51], and 50–70% have lymph node metastases at the initial study [94,110,113,114]. Lastly, 20–25% of all cases have ZES as part of the multiple endocrine neoplasia type 1 syndrome (MEN1) (ZES/MEN1) [115,116], and these patients are not curable without aggressive/extensive resections (Whipple procedures) because of the multiplicity of small duodenal primaries [108,109,113,117,118,119] frequently with lymph metastases [94,108,113,120]. Because of the excellent prognosis of MEN1/ZES patients with small gastrinomas (<1.5–2 cm), these more aggressive resections are not recommended routinely in most current guidelines [114,118,121,122,123,124,125].

Third, ZES patients, have a long-term disease course [110,120] allowing long-term observations of the effects of chronic hypergastrinemia. Not only is there usually a delay of 5–7 years to diagnosis [126], which is increasing because of increasing delays in diagnosis [18,38,127] and despite the fact that 60–90% of the gastrinomas are malignant [21,51] and <30% cured long term, the patients have an excellent long-term survival rate of 60–90% at 15 years after diagnosis [42,110,120] (Table 1). The long-term survival post diagnosis combined with the fact that the diagnosis is delayed 5–7 years after the disease’s onset [126] means that chronic hypergastrinemia will be present in majority of the patients longer than 25–30 years; hence, they provide very long-term follow-up of the chronic hypergastrinemic state. Now that the gastric acid hypersecretion can be controlled in all patients medically [28,31,34,35], the natural history of the gastrinoma is becoming an increasing determinant of long-term survival [114,118,120,121]; however, most patients continue to have extended survivals. This is the case because most gastrinomas are well-differentiated panNETs (G1/G2 grades, WHO 2017 classification), are relatively slow-growing compared to pancreatic adenocarcinomas [120,128,129], and despite not being cured, may be helped by surgical resection [93,107,130,131,132], as well as new antitumor treatments such as the use of somatostatin analogues, chemotherapy, molecular-targeted therapy, liver-directed therapies, and PRRT [128,133,134,135,136].

Fourth, studying ZES as a model of chronic hypergastrinemia does not have some of the limitations that studying other more common chronic hypergastrinemic states does, such as studying patients with chronic atrophic gastritis with or without Helicobacter pylori infection or with pernicious anemia [23,24]. Specifically, both of these latter conditions are associated with gastric inflammation, the development of gastric mucosal atrophy, and the presence of these correlates with the development of gastric carcinoids, and in fact, without their presence, ECL cell proliferative changes occur, but gastric carcinoid tumors are infrequent [2,25,73,74,77,137,138]. Therefore, the study of these latter conditions does not allow an uncomplicated study of the effects of chronic hypergastrinemia alone, whereas the situation with ZES more closely resembles what occurs with chronic PPI treatment. Helicobacter pylori (HP) infections can cause atrophic gastritis, and in patients with HP infections, the use of PPIs augments the mucosal inflammation and accelerates gastric mucosal atrophy, which may contribute to the development of gastric cancer [2,17,139]. In the US, the frequency of HP infections is 30–40%, therefore the majority of the US population, if treated with PPIs, will not have HP infection. Although HP infections are present in the majority of patients with duodenal ulcers not associated with NSAID usage (20,933), they are infrequent (<23–30%) in ZES patients [140,141,142], thus allowing effects of chronic hypergastrinemia without concomitant HP infection, inflammation, or atrophy to be studied.

Fifth, in ZES patients, studies of the reversibility of advanced gastric mucosal changes induced by chronic hypergastrinemia are possible [23]. This can occur because a proportion (up to 30%) of ZES patients can be surgically cured with resection of the gastrinoma. Curative resection results in both normalization of the fasting serum gastrin level as well as the positivity of the secretin-stimulated gastrin response [143,144] and a marked decreased in basal and maximal acid output [143,144]. 

Sixth, a proportion (20–25%) of ZES patients have it as part of the inherited autosomal dominant disorder MEN1 [115,116,145], which has a predisposition to the development of numerous endocrine tumors (parathyroid, pituitary, pancreatic endocrine, adrenal, gastric/thymic carcinoid tumors) [25,42,115,116,118,146]. This allows both the effects of chronic hypergastrinemia as well as the effects of PPI-induced changes to be studied in patients with an increased genetic predisposition to the development of neuroendocrine tumors.

Lastly, the study of ZES patients allows following patients during lifelong treatment with PPIs, with a significant proportion taking greater than the usual PPI doses taken by patients with non-ZES peptic/GERD disease (i.e., >equivalent omeprazole 20 mg/day) [22,28,30,31,35,147,148] (Table 1). The reason this occurs is because less than 30% of the ZES patients are cured long-term; thus, they require lifelong control of the gastric acid hypersecretion [21,29,34,149]. In the past, the only effective anti-secretory treatment was total gastrectomy [20,21,34,149], which resulted in few patients continuing to have intact stomachs, precluding any studies of the effect of chronic hypergastrinemia on the gastric mucosa. The development of effective medical control of acid hypersecretion in ZES patients, precluding the need for total gastrectomy, became possible with the development of first, histamine H_2_ receptor antagonists [52,98,150], and later by the late 1980s, the PPIs [32,33,34], which are now the drugs of choice in these patients because of their long duration of action, lack of tolerance, and continued efficacy when used once or twice a day [22,28]. This has resulted in the gastric acid hypersecretion of all patients with ZES being treated long-term with PPIs, and thus allowing long-term assessments of PPI’s possible long-term side-effects. 

## 6. Insights of Effects of Chronic Hypergastrinemia in Patients with ZES

### 6.1. Gastric Mucosal Effects in ZES Patients

#### 6.1.1. Gastric Mucosal Effects in ZES Patients; Non-Endocrine Cells 

Patients with ZES have increased gastric mucosal thickness [151,152] (i.e., 2-fold in one study) [152]. In ZES patients, there is an increase in the area of the fundus at the expense of the antral area [151]. The number of parietal cells computed as total number present [151,153,154], as a mean volume density in the lamina propria [155] or as the number of parietal cells per fundic gland [152], was higher in ZES patients than in controls. The total parietal cell mass in two studies [151] (4 ZES cases)/[156] (1 case) varied from 4 to 6 × 10^9^ cells with a mean of 4.6 × 10^9^ cells [151], which represented an increase in total parietal cell number of 4–6-fold above normal [151,153,156], an 8-fold increase [154], or 2-fold increased in a final study [152]. Another study [157] of a single ZES patient concluded the parietal cell mass calculated per unit volume of mucosa was increased 3-fold, whereas in another study of 50 ZES patients, no increase in the volume of parietal cells was detected; however, the total number of parietal cells was increased 4-fold [158]. The increased parietal cell population resulted from an increase in the area of fundus-type mucosa, an increase in the thickness of the mucosa, and an increase in parietal cell population per unit volume [159]. The total chief cell population varied from 4- to 10 × 10^9^ cells with a mean of 10.2 × 10^9^ cells (*n* = 4 cases) [151], which was proposed to be increased, although no controls were available [151]. A study directly examining the rate of gastric mucosal cell renewal in ZES patients [152] demonstrated a significant increase in proliferation of stomach epithelial cells, a faster cell generation rate owing to a reduction of the G1 phase by half, a 57% increase in the proliferative labeling index, and a broadening of the new cell generation zone from the bottom of the gastric pits in normal to the middle of the gastric pits in ZES patients, all supporting a marked proliferative effect of the chronic hypergastrinemia on the gastric mucosal cells in these patients. 

These data show that chronic hypergastrinemia in ZES patients, similar to reported in animal studies [151,160], has a trophic effect on the gastric mucosa, which results in both increased mucosal thickness, as was as increased parietal cell numbers.

#### 6.1.2. Gastric Mucosal Effects in ZES Patients: ECL Cells and Gastric Carcinoids

##### Gastric Mucosal Effects in Sporadic ZES Patients: ECL Cells and Gastric Carcinoids (Table 1)

**Figure 2:**
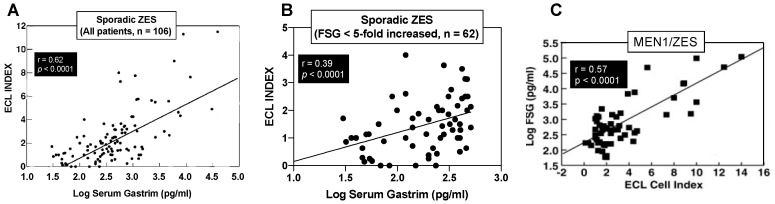
Correlations between the fasting serum gastrin levels (FSG) and the gastric enterochromaffin-like cells (ECL) cell proliferative index in ZES patients from two NIH studies. (**A**,**B**) show data from patients with sporadic ZES (*n* = 106) whereas (**C**) shows data from a study of patients (*n* = 57) with MEN1/ZES. All patients had multiple gastric biopsies and the proliferative ECL index was calculated from the degree of ECL changes in all biopsies and correlated with the FSG level. In (**A**) all 106 sporadic disease patients were included (90 active, 16 cured) and in (**B**), only patients with FSG levels <5-fold increased were included, which are levels overlapping with those seen in nonZES patients taking chronic PPIs. (**C**) shows the data from the 57 patients with MEN1/ZES. In all cases, there is a highly significant correlation of the FSG levels with the degree of ECL cell proliferative change. In (**B**), the data shows there is no threshold for gastrin’s ability to stimulate ECL cell proliferative effects as was proposed in the past. Figures are drawn from the data in [25,61].

Numerous studies report increased gastric ECL proliferative changes in different series of ZES patients [25,36,47,61,71,72,155,158,161,162,163,164,165,166,167,168,169]. As discussed below there are marked differences in the response to chronic hypergastrinemia in gastric ECL cells in patients with MEN1/ZES and sporadic ZES, and many of the above series contain mixtures of these patients. The largest study dealing with only sporadic ZES patients was an NIH study [25] included 106 patients (90 active, 16 cured) in which all patients with active disease had chronic hypergastrinemia and the disease was long-standing with mean duration of ZES since onset at the time of the study of 13 years and a median fasting gastrin level 4-fold elevated. Gastric ECL cell hyperplasia was found in 99% of the patients, 50% had advanced changes, and 7% showed dysplasia [25]. The level of hypergastrinemia correlated directly with the degree of ECL cell change (*p* = 0.057, *p* < 0.0001) [25] as shown in Figure 2A. Increased ECL changes also correlated with cure status, the duration of drug treatment, and the presence of atrophic gastritis, but not gender [25].

**Figure 3:**
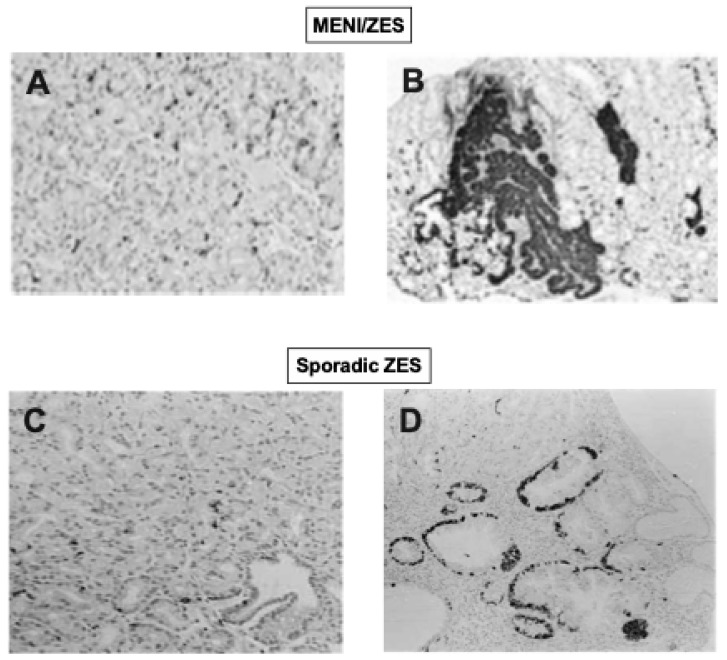
Examples of gastric ECL cell proliferative changes in ZES patients. In (**A**,**B**), results from gastric biopsies in two patients with MEN1/ZES are shown. In (**A**), a normal distribution of chromogranin A positive ECL cells in the oxyntic mucosa is shown. (**B**) shows a second MEN1/ZES patient a small intramucosal ECL-cell tumor (on the left), in association with an ECL-cell dysplastic lesion (in the center) and severe LH of ECl cells (on the right). In (**C**,**D**) are results from patients with sporadic ZES. (**C**) shows a normal ECL pattern with chromogranin staining and (**D**) shows micronodular and linear hyperplasia of ECL cells (in black) in a biopsy specimen of a patient with ECL cell dysplasia. Pictures are from Prof. C. Bordi (Univ. Parma) and made from data in [25,61]. Advanced ECL changes occurred in 53% of patients with MEN1/ZES with 2% showing dysplasia and 23% of patients have a gastric carcinoid found [61]. In sporadic ZES patients, 50% of patients had advanced ECL cell changes, 7% showed dysplasia, and none had a gastric carcinoid. The latter data show that advanced ECL changes including dysplasia can occur with prolonged chronic hypergastrinemia in man (mean duration of 13.2 yrs.) without any other contributing features such as the presence of MEN1, gastric mucosal atrophy, or gastric mucosal inflammation. 180X.

The finding that all sporadic ZES patients (80–100%) had at least one gastric mucosal biopsy showing ECL cell diffuse/linear hyperplasia or more advanced ECL changes agreed with results from other small studies in patients with sporadic ZES [62,161,165,166,167,170,171], but differed from results in one study, which reported only 14% of ZES patients had such ECL changes [158]. The results in the NIH study [25] demonstrating 11% of sporadic ZES patients had micronodular hyperplasia and 7% dysplasia (Figure 3, bottom panels), differ from other studies of patients with sporadic ZES reporting no patients were found to have either of these advanced ECL cell changes [36,158,165,171]. The results of the above NIH study [25] demonstrating the presence of advanced ECL cell changes in some ZES patients refutes the proposal, from the lack of advanced ECL cell changes found in three studies of ZES patients [36,158,165], that chronic hypergastrinemia in man alone was not sufficient to induce advanced ECL cell changes without the contribution of another risk factor such as MEN1, atrophy, inflammation, or gastritis [25].

An important finding from the above NIH study [25] on sporadic ZES patients was revealed from an analysis of the correlation of the elevation of FSG at the time of the gastric biopsies with the most advanced ECL cell change found, which allowed insight into whether the gastrin-induced proliferative effects on ECL cell proliferation has a threshold effect. It has been proposed from a number of studies on non-ZES patients that because of a failure to see ECL cell proliferative changes with low elevations of FSG, that a threshold effect for proliferative effects of increased FSG existed in humans that is 4–5-times the upper limit of normal [47,61,172,173]. In the above NIH study of sporadic ZES patients [25], this was found not to be the case with a direct correlation found between increasing fasting gastrin levels and the degree of ECL cell proliferative changes. An analysis of this data including patients with FSG ≤ 5 times increased (≤500 pg/mL) in that study (Figure 2B) demonstrates that there is a highly significant direct correlation between any increase in FSG and the degree of increase in the ECL proliferative change without any apparent threshold effect. The conclusion that there is no threshold effect for gastrin for inducing ECL cell proliferative changes in humans is consistent with results of two other humans’ studies: One involving patients with gastric acid hypersecretory states treated with lansoprazole [169] and the other with results from a study of patients with atrophic gastritis [25,174]. 

In the above study involving 106 sporadic ZES patients [25], no patient was found to have a gastric carcinoid tumor. This result agrees with a number of other studies of sporadic ZES patients in which no gastric carcinoid tumor was found [36,62,158,161,166,167,168,175]. This result is in contrast to a few case reports of patients with sporadic ZES who had gastric carcinoid tumors found [23,47,71,72,138,171,176,177,178,179,180]. These results show that, despite prolonged chronic hypergastrinemia averaging 14 years in the large NIH study reviewed above [25] and the fact that the ZES patients in more than half of the cases had high fasting serum gastrin (FSG) levels at least double that seen typically in patients being treated with PPIs, with many ZES patients with FSG > 5–10 levels (Figure 1), the occurrence of gastric carcinoids in the sporadic ZES patients was very uncommon. This occurred even though advanced ECL cell changes occurred in up to 11% and dysplasia in 7% of sporadic ZES patients in one study [25]. These results are in marked contrast to the results with patients with chronic atrophic gastritis where the average gastrin levels are usually less than seen in many ZES patients [163], however 0.4–7% of all chronic atrophic gastritis patients have gastric carcinoids on routine endoscopy, with rates varying from 5–35% in some series with long-term follow-up [60,75,181]. 

At present, the basis for this discrepancy in the frequency with which sporadic ZES patients and chronic atrophic gastritis/pernicious anemia (CAG/PA) patients develop carcinoid tumors is unclear, although previous studies suggest a number of factors that could be important. First, in studies in rats treated long-term with omeprazole, the development of advanced ECL cell changes, as well as the frequency of carcinoid tumors, was very much dependent on the duration of drug treatment, as well as showing a late life augmentation [46]. Similarly, in some studies of CAG/PA patients, the duration of the disease is reported to be a factor in the development of the gastric ECL cell tumors [182,183]. This could be a major contributing factor to the difference in frequency with which ZES patients and CAG/PA patients develop gastric carcinoids. This could occur because in many patients it is unclear exactly when the CAG/PA began, and especially in older studies, if *Helicobacter pylori* was unknowingly present and possibly functioning as a contributing factor to the CAG development, the CAG could have been present much longer than thought. In the case of ZES, the onset of the disease is usually taken as the onset of consistent peptic/GERD symptoms [126,184] so that the duration of the disease can be better defined. The possible importance of duration of disease as an important contributing factor is supported by results in patients with ZES/MEN1, which will be discussed in detail in the next paragraph, which shows that ZES disease duration is an important factor correlating with the development of gastric carcinoids in these patients [61]. In fact, in one study [61] of MEN1/ZES patients, it was reported that there was a 27% increase in the rate of development of gastric carcinoids for each additional 10 years of the disease. 

A second possible major factor that could be contributing to the marked difference in rate of development of gastric carcinoids in patients with sporadic ZES or CAG/PA, is the frequency of atrophy of the gastric mucosa, mucosal inflammation, or gastritis with or without *H. pylori*, all of which have been shown to be associated with the development of more advanced ECL changes and gastric carcinoid tumors in CAG/PA patients [25,73,74,137,138,185]. In contrast, ZES patients uncommonly have chronic atrophic gastritis; in fact, in most cases, the mucosa is hyperplastic [25,126,151,152]. Furthermore, in ZES, only 23–44% have *H. pylori* infections [140,141,186] and in only 27–33% is moderate/advanced gastritis present [25,186], thus they do not have these associated factors present to promote the development of the gastric carcinoids in most patients, which is not the case in chronic atrophic gastritis. 

A third factor that could also be contributing to the frequency with which gastric carcinoids are reported in ZES patients is the limited number of ZES patients with intact stomachs available for study until the last decade. Prior to the availability of PPIs in the late 1980 to the early 1990s, one of the most frequently used acid anti-secretory treatments was total gastrectomy [20,28,34]. Even though histamine H_2_ receptor antagonists were available in the 1980s and shown to be effective if used according to strict criteria, in most patients, high, frequent dosing was required, which could not be predicted in a given patient, thus the dose had to be individually titrated in each patient using an established criteria of acid suppression (<5–10 mEq/hr prior to the next drug dose) [28,34,52,53,187,188,189]. This was not done in many centers and the medical failure rate was high in many series [28,34] with the result that total gastrectomy continued to be used in many patients, until PPIs became available and could control acid hypersecretion in every case with once or twice a day dosing [28,30,31,32,33,147].

##### Gastric Mucosal Effects in MEN1/ZES Patients: ECL Cells and Gastric Carcinoids (Table 1)

As pointed out above, 20–25% of ZES patients have it as part of the autosomal dominant disorder MEN1 [115,145,190], which is due to a mutation in the 10-exon MEN1 gene on chromosome 11q13, which encodes for a 610 amino acid protein, menin, which is a nuclear protein interacting with numerous transcription factors involved in genomic stability, transcriptional control, cell division, and cell cycle control [115,190]. ZES develops in 20–61% (mean—54%) of MEN1 patients at some time [115,116,118]. In addition, MEN1 patients have an increased predisposition to develop numerous other endocrine, as well as nonendocrine tumors tumors (parathyroid > pancreatic endocrine > pituitary > adrenal > gastric/thymic carcinoid tumors, tumors of smooth muscle, and skin) [25,42,115,116,118,146]. ZES in MEN1 patients presents a decade earlier than in sporadic ZES cases [116]. MEN1/ZES patients are rarely cured because >85–100% have multiple, small duodenal gastrinomas, which metastasize to lymph nodes early, and without aggressive resections such as a Whipple procedure, are not cured by simple tumor resection or tumor enucleation [115,116,118,121,122,123]. Because they have an excellent prognosis with small gastrinomas and/or with NF-panNETs (<2 cm), most guidelines (ENETs, NANETs) do not recommend aggressive resection unless the panNETs >2 cm or show aggressive growth [115,118,121,122,123,124]. Therefore, the combination of early onset of ZES in MEN1 patients, combined with lifelong chronic hypergastrinemia and mutations in the MEN1 gene predisposing them to the development of endocrine tumors, these patients not only very frequently develop advanced ECL cell changes, but also gastric carcinoids, which are more aggressive than type 1 sporadic gastric carcinoids in CAG/PA or other hypergastrinemia states [42,61,115,116,121]. 

Numerous studies report that MEN1/ZES patients not only can developed advanced stages of ECL cell proliferative changes (0–33%), but in contrast to sporadic ZES patients [25], they also frequently (10–30%) develop gastric carcinoids [61,62,77,116,159,161,165,166,177,184,191,192,193,194,195,196,197,198,199]. The most complete data in one study come from a large NIH prospective study involving 57 consecutive patients with MEN1/ZES [61], in whom at the time of the gastric biopsies, the ZES had been present a mean of 11.4 years (range, 0.5–31.4 years), and the patients had all had chronic hypergastrinemia at the time of the biopsy with the FSG elevated a mean of 4.7-fold above normal (range—1–1100-fold increased). All of the 57 patients in this study [61] were found to have ECL cell proliferative changes and the degree of the proliferative change highly correlated with the FSG levels (Figure 2C). In 53% of the MEN1/ZES patients in this study, advanced proliferative changes were found (Figure 3, top panels), and 23% had a carcinoid tumor. Risk analysis demonstrated that gastric carcinoid tumors were more frequent in MEN1/ZES patients with higher FSG levels, longer disease duration, and strong alpha-HCG staining on the biopsy [61]. Multivariate analysis demonstrated that for each 10-fold increase in FSG level, there was a 67% increase in the ECL cell proliferative index, which was greater than the effect of ZES disease duration, which resulted in a 27% increase in the ECL cell proliferative index for each 10 years of increased ZES disease duration [61]. The universal frequency of the gastric ECL proliferative changes found in this large perspective study of MEN1/ZES patients [61] is similar to results with other smaller studies (usually < 20 MEN1/ZES patients), which also reported 92–100% of MEN1/ZES patients had such changes [62,163,164,165], but differ from other small studies, which reported these changes in an only 14–65% of MEN1/ZES patients [166,200]. Also, the finding of 23% of the patients having gastric carcinoids is a higher rate than reported in the literature in MEN1/ZES patients in most (0–14% (mean 2 ± 1%, from 9 series), but not all (25–33%) small series [36,61,62,116,163,165,166,169]. The higher rates of detection in the NIH perspective study were at least partially related to the fact that all patients had large biopsies taken with Jumbo forceps from four fixed areas of the greater and lesser curvatures and two from the antrum for a minimum total of 10 biopsies, with any gastric nodules or suspicious areas biopsied additionally. A previous study [47] of the effect of biopsy location and number on the ability to diagnose ECL proliferative changes and identify gastric carcinoids or dysplasia in ZES patients showed the frequency of finding a gastric carcinoid or gastric ECL cell dysplasia detection was directly related to biopsy number, with eight biopsies required to detect all the carcinoid tumors/dysplastic lesion found. Furthermore, the location of the gastric biopsy influenced whether the most severe gastric ECL proliferative changes were detected [47]. 

##### Gastric Mucosal Effects in ZES Patients: ECL Cells and Gastric Carcinoids-Sporadic ZES versus MEN1/ZES 

The data both from the two large NIH studies reviewed above on gastric ECL changes in sporadic ZES [25] and MEN1/ZES [61] patients, as well as from the literature, demonstrate that chronic hypergastrinemia in MEN1/ZES patients is associated with more advanced ECL cell proliferative stages the seen in sporadic ZES patients, as well as a marked increase in the occurrence of gastric carcinoid tumors. From a review of the data in these studies, it was calculated that gastric carcinoid tumors are at least 70-fold more frequent in MEN1/ZES patients that in sporadic ZES [61]. This difference in the two NIH studies was not due to a difference in duration of the hypergastrinemia, because the ZES duration at the time of the gastric biopsies was similar in the two studies, nor was it due to differences in the level of the FSG in the two groups, which was similar [61]. These results support the proposal that in MEN1/ZES patients the presence of their MEN1 gene mutation and its subsequent cellular effects are responsible for the increased gastric mucosal proliferative effects of the chronic hypergastrinemia [201,202]. 

##### Gastric Mucosal Effects in ZES Patients: Comparison with Results from Studies of Chronic Hypergastrinemia in Animals, ECL Cell Changes Reported with Chronic PPI/Potent Anti-Secretory Drug Use, and ECL Cell Changes in Other Common Chronic Hypergastrinemic States (CAG/PA) in Man

The results of the reported effects of chronic hypergastrinemia on gastric ECL cells and other gastric mucosal cells from both groups of ZES patients (i.e., sporadic ZES and MEN1/ZES) show a number of close similarities, as well as some differences with the results from studies of the gastric mucosal effects of chronic hypergastrinemic in animals, as well as seen with chronic use of PPIs in humans and other common chronic hypergastrinemia disorders in man such as chronic atrophic gastritis/pernicious anemia (CAG/PA). 

Similarities between the gastric mucosal effects of chronic hypergastrinemia in ZES and in animal studies include a number of observations. In dog, rat, and mouse, long-term PPI treatment resulted in an increase in oxyntic mucosal thickness and folding [203], similar to that seen in both groups of ZES patients. With animal studies, the degree of the chronic hypergastrinemia induced by PPIs, other anti-secretory drugs, or other methods, correlated directly with the extent of the ECL cell changes, as it does in both sporadic ZES and MEN1/ZES patients [25,56,61,62,204]. Long-term treatment in rats with either PPIs or the insurmountable H_2_ antagonist, loxtidine, resulted in advanced ECL changes and gastric carcinoids [203,205], whereas in mice, chronic PPI treatment caused advanced ECL changes, but no gastric carcinoids [203], similar to that seen in sporadic ZES. In contrast, long-term loxtidine treatment in mice caused both advanced ECL changes and the development of gastric carcinoids [206]. 

A number of differences between the gastric endocrine cell effects of chronic hypergastrinemia in patients with ZES and from animal studies are also evident. In studies in rats, female gender was associated with more advanced ECL cell proliferative changes and rate of development of gastric carcinoids than seen in male rats with chronic hypergastrinemia induced by various antisecretory drugs (with PPI, other anti-secretory treatments) [203,205,207]. In contrast, chronic treatment with the insurmountable H_2_ antagonist, loxtidine, causes gastric carcinoids primarily in female rats, but equally in male and female mice [205,206]. These results differ from those of the large NIH studies on both sporadic ZES and ZES/MEN1 where gender was not a factor either in the severity of the chronic hypergastrinemic induced ECL changes, nor in the development of gastric carcinoid in MEN1/ZES patients [25,61]. However, in a few other small studies of ZES patients, increased ECL cell proliferative changes/gastric carcinoids were more common in females in some [74,161,165] but not in other studies [158].

The gastric endocrine cell changes in patients with CAG/PA were similar to those seen in ZES with the extent of the ECL cell changes also directly correlated with the degree of hypergastrinemia in both [62,76,208,209,210,211]. With both CAG/PA and both forms of ZES, advanced ECL cell changes can occur including dysplasia, however only MEN1/ZES demonstrates the frequent occurrence of gastric carcinoids, similar to reported with long-term CAG/PA, and thus these differ from sporadic ZES, in which gastric carcinoids are very rarely reported.

There are also a number of important differences between the gastric endocrine cell changes in CAG/PA and other chronic hypergastrinemic states in humans compared to ZES. In ZES patients, it has been shown that of the six types of gastric endocrine cell types, only the ECL cell fraction is increased with chronic hypergastrinemia, demonstrating the specificity of the gastrin-trophic effect on these cells [167]. In contrast, in patients with chronic hypergastrinemia with CAG/PA, in addition to the ECL cells, the P cells and the D_1_ cells are also increased [47,176]. Another important difference is the effect of gender differs between the two diseases with a number of studies reporting females have a high incidence of advanced ECL changes and/or carcinoids with various other human chronic hypergastrinemic states (CAG/peptic ulcer disease) [208,212,213], whereas it did not matter in the two NIH ZES studies [25,61]. Another prominent difference is the relative importance of chronic atrophic gastritis in patients with ZES or CAG/PA. Atrophic gastritis is uncommon in sporadic ZES patients (<15%) [25], however its presence in these small number of ZES patients is associated with more advanced ECL proliferative changes [25]. In contrast, in patients with CAG/PA, atrophic gastritis is present and it plays an important role in the both the extent of ECL cell proliferative changes and in the development of carcinoid tumors, which in both cases, its presence is an independent risk factor [209,214].

In patients taking long-term PPIs or other potent gastric acid suppressants, the gastric mucosal changes also show many similarities to the findings in ZES patients, particularly in patients with sporadic ZES. In both groups of patients, the extent of ECL cell changes correlated directly with the extent of increase in the circulating gastrin levels [25,215]. In both groups of patients, ECL cell proliferative changes frequently occur. Similar to the case with sporadic ZES, reports of patients treated long-term with PPIs or other potent gastric acid suppressants (without ZES, CAG), developing gastric carcinoids thought due to the PPI is very uncommon [67,68,69,70,216,217,218]. Similar to sporadic ZES the ECL proliferative changes primarily include diffuse, linear, and micronodular hyperplasia and the presence of dysplasia is absent/uncommon in patients with long-term treatment PPIs or other potent gastric acid suppressants [215].

A number of differences have been reported due to the chronic hypergastrinemic effects on the gastric endocrine cells in patients with chronic ZES and those found in patients taking long-term PPIs or other potent gastric acid suppressants. These differences include the importance of female gender showing more advanced ECL cell changes with chronic PPI treatment or with H_2_-receptor antagonists [215], whereas it was not an important variable for ECL cell changes in the NIH sporadic ZES study [25] or in the ZES/MEN1 study [61] study. Another important difference is the prominent role of atrophic gastritis and *H. pylori* infections in the two groups of diseases. *H. pylori* is relatively uncommon in the ZES and its presence in sporadic ZES patients did not correlate with the severity of the ECL cell proliferative changes [25]. In contrast, in non-ZES patients taking long-term PPIs or other potent gastric acid suppressants, the presence of *H. pylori* was an independent risk factor for both increased ECL cell density and the degree of ECL cell proliferative change [215]. Atrophic gastritis is also relative uncommon in sporadic ZES patients (<15%) [25], but its presence in ZES is associated with more advanced ECL proliferative changes. In one study of non-ZES patients treated with long-term PPIs or other potent gastric acid suppressants, in those treated for GERD, 30–40% have *H. pylori*, and 50% have atrophic gastritis [215]. In these patients, the presence of the atrophic gastritis correlates strongly with the presence of *H. pylori*, and the presence of the atrophic gastritis is an independent predictor of the presence of ECL cell proliferative changes [215]. In some [139,219] but not all [215,220] studies of GERD/PUD patients, long-term treatment with PPIs or other potent gastric acid suppressants is associated with an increase in the incidence/severity of the atrophic gastritis in *H. pylori*-positive patients, which can correlate with degree of the ECL cell proliferative changes. The result of these changes with long-term anti-secretory drug treatment with GERD/PUD is that the severity of the gastritis/atrophic gastritis plays a major, independent role in the severity of the ECL cell changes, which is usually not the case in the ZES patients.

In 1992 [74], Prof. Solcia in a review of ECLomas and their growths stated that the evidence supports the conclusion in man the gastrin alone is able to “promote the proliferation of ECL cells but is per se apparently unable to induce ECL transformation.” A number of more recent reviews have concluded similarly. The data reviewed here from the long-term NIH ZES studies, as well as the data from smaller studies of sporadic ZES patients, which are most like non-ZES patients taking PPIs long-term for GERD/PUD, can be interpreted as generally supporting the proposal of Prof. Solcia. However, some important reservations still seem justified.

First, while no gastric carcinoids were seen in the sporadic ZES patients in the NIH study and are very uncommon in the literature also in these patients, we did see dysplasia, which is proposed to be the most advanced ECL cell proliferative change that leads to the development of gastric carcinoids [74]. 

Second, even the NIH ZES studies can be considered short term as the mean follow-up is slightly longer than a decade, which is not a lifetime, as could be case with lifelong PPI treatment. Data from the NIH MEN1/ZES study [61] as well as from animal studies [46] and studies of atrophic gastritis/PA [182,183] support the importance of disease duration for the development of advanced ECL changes. The fact that much longer time periods of chronic hypergastrinemia alone coupled with continued achlorhydria may have an effect that is not mirrored in the studies reviewed above is supported by a recent description of five children from consanguineous parents [50]. These five siblings had an inherited defect in the H^+^K^+^ ATPase gastric proton pump gene (mutation in ATP4A gene) and thus had chronic hypergastrinemia from birth. However, it was only at ages 23–39 that they had gastric carcinoids diagnosed, which were aggressive requiring total gastrectomy in three [50]. Therefore, it can not necessarily be concluded from studies of <5 years in most cases or even up to 10 years, the much longer exposure to chronic hypergastrinemia may not result in more advanced changes in ECL proliferation with gastric carcinoid development. 

Third, numerous studies show that not only *H. pylori* infections, but also the degree of gastritis can play a major role in the extent of development of advanced ECL cell changes in different models of chronic hypergastrinemia. The frequency of these two parameters can vary markedly in different populations and have a marked effect on the ECL changes, which are not well reflected in the long-term ZES studies, because ZES patients have generally a lower frequency of gastritis, the gastritis if present is generally mild, and *H. pylori* infections are less frequent.

Fourth, numerous recent studies have supported the safety of long-term PPI treatment in terms of lack of development of gastric carcinoids, but even in these studies the follow-up is less than a decade. 

### 6.2. Other Effects of Chronic Hypergastrinemia in ZES

In addition to the well-established effect of gastrin to stimulate the proliferation of gastric ECL cells, which was reviewed above, numerous studies in animals, as well as in vitro studies, have reported that either gastrin or its precursors can have important growth/development effects on a number of other neoplasms. These include particularly roles in the development or stimulation of esophageal cancers, gastric cancer, pancreatic cancer, and colon cancers [78,79,81,82,84,221,222]. At present, the role of gastrin-related peptides in the growth or development of any of these cancers in humans is controversial and unclear.

#### 6.2.1. Other Effects of Chronic Hypergastrinemia in ZES: Gastrin and Esophageal adenocarcinoma (Table 1)

In the last three decades, the risk of esophageal adenocarcinoma (EAC) in the US has increased >6-fold [222]. It is well-established that Barrett’s esophagus (BE) is a precursor lesion that increases the risk of EAC, which in some studies, the risk of developing EAC with BE is increased >30-fold (20,914, 20,774). This increased rate of EAC coincides with the increased use of potent gastric acid suppressants, particularly PPIs [223]. The role of chronic hypergastrinemia in the development of EAC remains controversial and unclear similar to the possible role/use of chronic use of PPIs [223,224,225]. Studies show that BE cells express CCK2R (gastrin) receptors, that gastrin can stimulate the proliferation of BE cells, whereas other studies report gastrin can promote the development of BE and recent data suggest that BE may originate from gastric stem cells which also possess CCK2R receptors [223,226,227]. However, the role of gastrin or PPI in the progression to EAC is controversial because some studies [225,228,229,230], but not others [225,231] report that long-term PPI treatment may decrease BE progression and be beneficial in BE patients. In contrast to the role of PPIs in BE, a recent epidemiological study of 797,067 subjects reported the chronic use of PPIs increased the risk of developing esophageal adenocarcinoma, but not squamous adenocarcinoma of the esophagus, and the risk remained 91% increased after five years of PPI use [85,232]. 

In patients with ZES, there are no data to suggest an increased occurrence of EAC from case reports or individual series, however this conclusion is based on limited systematic studies. In a prospective study of 261 ZES patients from the NIH [126], 42% of patients with sporadic ZES and 52% of MEN1/ZES patients had esophageal symptoms (primarily heartburn) at initial presentation [126], which compares to 29–61% in other series [116,126,147,233,234,235,236], demonstrating that chronic GERD symptoms are not infrequent in these patients. Furthermore, evidence for severe GERD is not infrequent in ZES patients with severe GERD symptoms frequently present, grade 3 esophagitis found frequently (23%), as well as esophageal strictures due to chronic GERD (10–13%), all reported in a significant number of ZES patients [126,147,233,236]. However, the frequency of BE is reported in only 3–6% of ZES patients in various series composed primarily of sporadic ZES patients [233,237], which is lower than the mean frequency of 23% (range 5–44%) in patients with moderate to severe idiopathic GERD and the 56% reported in patients with chronic GERD symptoms with idiopathic gastric acid hypersecretion [237,238]. This relatively low frequency of BE in ZES patients despite the fact that they have chronic basal acid hypersecretion 4–8-fold higher that either idiopathic GERD patients or patients with idiopathic hypersecretion, combined with the finding that most ZES patients have normal esophageal motility [237], has led to the suggestion that ZES patients may have other protective factors such as increased EGF or bicarbonate section contributing to increased esophageal mucosal resistance to the high gastric acid levels [237]. One study supports this later proposal because patients with ZES were found to have increased EGF concentrations in both their saliva and basal gastric fluid [239]. MEN1/ZES differ from sporadic ZES patients in having the ZES present at an earlier age and frequently having a higher frequency of patients whose acid hypersecretion is under-treated [236]. The latter point likely contributes to the finding in one large comparative study of esophageal disease in the two groups of ZES patients [236], that the MEN1/ZES patients had a 3-fold higher incidence of esophageal stricture, a 5-fold higher BE occurrence, 8-fold higher development of dysplasia, and one MEN1/ZES patient developed an esophageal adenocarcinoma, whereas none were seen in 315 sporadic ZES patients who had been followed for a mean of 14 years.

Besides the patient with MEN1/ZES discussed in the above paragraph who developed an esophageal adenocarcinoma, in the literature, there are no other reports of patients with EAC with ZES. In a review of the prognosis and survival in 1716 patients with ZES/MEN1 in the literature, no patients were reported to have an esophageal adenocarcinoma [42]. In another review of 758 MEN1 patients of which 23% had ZES, no patient died from an esophageal adenocarcinoma [240]. While these data do not suggest an increased incidence of esophageal adenocarcinoma in ZES patients, there are a number of deficiencies that limit the strength of the conclusion. First, the rate of esophageal adenocarcinoma in the US is 2.58/100,000 in the general population [241] and the long-term survival data are only available on a limited number of ZES patients (<3000–5000), therefore even a moderate increase incidence in EAC rate could easily be missed. Second, although ZES patients are now living longer, overall it is still shortened for many patients [110,120], and MEN1 patients still have a much-shortened overall survival (mean death—55 years) [42], therefore extended long-term follow-up is from an even smaller pool of ZES patients. 

#### 6.2.2. Other Effects of Chronic Hypergastrinemia in ZES: Gastrin and Gastric Adenocarcinoma (Table 1)

The role of chronic hypergastrinemia in the development of gastric carcinomas (GC) is also controversial [79,87,223]. A particularly important predisposing factor for the development of GC is the presence of gastritis which worldwide is closely related to the presence of *H. pylori* infection [79,87,218,223] and a recent meta-analysis concluded that eradication of *H. pylori* was reducing the risk of GC [242]. Furthermore, autoimmune gastritis such as that that occurs in pernicious anemia, also predisposes one to the development of GC [76,79]. Gastritis increases the risk of GC when it effects the oxyntic mucosa of the stomach, principally when its presence results in the development of oxyntic atrophy and with time the development of intestinal metaplasia [79,223]. More recent studies demonstrate that treatment with PPIs resulting in reduced acidity leads to the migration of *H. pylori* from the antrum to the corpus resulting in an increased colonization of the gastric corpus, which is associated with the development of corpus inflammation, gastritis, and an increase risk of developing atrophic gastritis [139,243,244]. The atrophy of the corpus area resulting in chronic hypergastrinemia has been shown to be a significant risk factor for the development of GC [244,245]. The role of chronic hypergastrinemia in development of GC was further supported by studies on insulin-gastrin (INS-GAS) transgenic mice [223]. INS-GAS mice have chronic hypergastrinemia, which initially results in increased acid secretion with parietal cell proliferation, but later gastric metaplasia develops with dysplasia, and finally GC [223,246]. Furthermore, infection of the INS-GAS mice with *H. pylori* accelerated the development of the GC and the GC development was markedly inhibited by the addition of a CCK2R (gastrin) receptor antagonist [247]. In addition to gastrin’s role in the development of GC, numerous studies report that gastrin may directly affect GC proliferation, frequently in an autocrine fashion [83]. GC’s frequently overexpress CCK2R (gastrin receptors), as well as ectopically express the gastrin gene [83,248], and both amidated, as well as progastrin and gastrin processing intermediates, are reported in GCs [83,248] supporting the presence of an autocrine growth loop [83,248]. Another proposed pathway that gastrin may contribute to the development of GCs is through the stimulation of ECL cells [11]. ECL cell proliferation and GCs are often present together and a number of studies have provided evident to support the proposal that the diffuse form of GC can originate from the ECL cells [11]. In female cotton rats treated with the insurmountable histamine H_2_ receptor antagonist, loxtidine, GC develops and in the dysplastic mucosa, positive staining for ECL cell markers can be found, suggesting an ECL cell origin of the GC tumors [49]. A number of large epidemiological studies have recently provided evidence that prolonged chronic PPI use is associated with an increased rate of gastric cancer [85,86,249]. In one study, which included 63,397 individuals from Hong Kong [249], the use of PPIs was associated with a HR of 2.24 for developing gastric cancer, while the use of histamine H_2_ receptor antagonists was associated with no increased risk. The risk increased with longer PPI use to HR 8.34 at ≥3 years. In a second study of 797,067 individuals in Sweden, chronic PPI use was associated with a 48% increase in gastric cancer after one to three years use of PPIs and a 31% increase after five years use [85,86]. 

Studies of patients with ZES provide no evidence for an association of chronic hypergastrinemia in these patients and the presence or development of GC. No death due to GC or even any cases of GC in ZES patients have been seen in the NIH studies (>350 ZES patients). Similarly, no cases of GC are reported in 1716 patients with ZES/MEN1 in the literature in one review of survival in these patients [42] or in another review of risks of death in 758 MEN1 patients of which 23% had ZES, no patient had GC [240]. There are occasional case reports of carcinomas of stomach in patients with ZES [250,251]. In two cases, gastric collision tumors were described in patients with ZES with co-existence of a separate gastrinoma and adenocarcinoma [250,251]. In another case, a patient with a duodenal gastrinoma and ZES/MEN1 with gastric ECLomas was found to have a signet-ring carcinoma of the stomach [252]. Similar arguments to those reviewed above for failure to detect an association with chronic hypergastrinemia and esophageal cancer in ZES patients can be applied for the failure to see a similar association with gastric cancer in ZES patients. Another important variable that could affect this association in ZES is the low incidence of both *H. pylori* and atrophic gastritis in ZES patients, which are important predisposing factors for GC. Whether the long-term use of PPIs in these patients will change the incidence of gastritis or the effect of *H. pylori* in some of these patients is, at present, unclear. This could occur because studies show that PPIs can essential render many ZES severely hypo-/achlorhydric [27,31,147,148,253].

#### 6.2.3. Other Effects of Chronic Hypergastrinemia in ZES: Gastrin and Colorectal Cancer (CRC) (Table 1)

Numerous in vitro studies, studies in animals, as well as findings in human colorectal cancers (CRCs) and other human studies, have all provided evidence that gastrin-related peptides (primarily progastrin and processing intermediates (gastrin precursors)) may play a role in CRC pathogenesis [78,224,254,255]. Growth effects of gastrin precursors (non-amidated) have been demonstrated in normal colonic tissue in both in vitro and in vivo studies [78,254,255]. In studies of gastrin-knockout mice, a reduced proliferative index was found in the colonic mucosa and an infusion of gastrin–glycine-extended peptide, but not amidated gastrin, resulted in an increase in the proliferative index in these mice [255]. In a non-transformed colon cell line, glycine-extended gastrin functioned as an autocrine growth factor [256]. In other studies, glycine–extended gastrin (Gly-Gastrin) is reported to have proliferative effects on the colonic mucosa in mice overexpressing Gly-Gastrin; and similar effects are seen in transgenic mice over-expressing progastrin, which in the latter case, in the presence of p53 mutations, CRC is seen [254,255]. Furthermore, the autocrine secretion of progastrin is reported to promote the survival and self-renewal of colon cancer stem cells [257]. At present, exactly what receptor(s) mediates the actions of the progastrin or Gly-Gastrin and whether the classical CCK2R (gastrin receptor for amidated gastrin) is involved in the above actions is not clear [254]. Studies reporting an increased frequency of CRC in human hypergastrinemic states (CAG/PA, chronic PPI use) or studies of a correlation between the serum gastrin levels and frequency of CRC where serum amidated gastrin was assessed have given variable results, with most recent studies reporting no correlation [78,258,259,260,261,262]. Two studies assessing serum gastrin precursors have reported elevated levels in patients with CRC [263,264].

The above results, suggesting that circulating gastrin precursors could be of particular importance in the pathogenesis/growth of CRC, should make the study of ZES an attractive model to study their role in CRC, because numerous studies have demonstrated that patients with gastrinoma have high circulating levels of not only amidated gastrin, but also progastrin, amino-, and COOH terminal gastrin fragments, as well as Gly-Gastrin [21,39,265]. In the literature, there are only a few cases describing the presence of CRC in a patient with ZES [266,267]. In a review of survival of MEN1/ZES patients at both NIH (*n* = 106), two (1.9%) patients had CRC, and in 223 patients from the literature, two (0.88%) patients died from CRC [42]. Furthermore, of 1603 MEN1 patients in the literature of which 23% had ZES, only eight patients (0.005%) died from CRC [42]. One prospective study [26] of 97 consecutive ZES patient all of who had colonoscopy successfully reaching the cecum in which the mean FSG was 31-fold elevated with a mean disease duration of 10 years, 17/97(18%) had adenomatous polyps and 2/97(2%) had CRC. These rates were within the ranges reported in non ZES patients or autopsy studies when stratified for age or gender [26]. In another study [268] of 23 consecutive patients with ZES (six with MEN1/ZES), 5/23 (22%) of patients were found to have a colonic adenoma, which was a rate thought not to be different from the general population for patients of comparable age (all > 50 years old) [268]. The proliferative rate was assessed in these patients and normo-gastrinemic controls by determining in vitro 5’-bromodeoxyuridine labeling from two biopsy sites in the colon [268]. The labeling indices were significantly higher in the ZES patients than in 18 normogastrinemic controls both in the right colon (*p* < 0.2) and the left colon (*p* < 0.001) [268]. No colonic cell hyperplasia was seen, and the DNA labeling distribution was normal in the ZES patients without any expansion of the preoperative zone [268]. In a third study [269], the rectal mucosal cell proliferative rate was compared in patients with ZES (*n* = 6), CAG (*n* = 10), and controls (*n* = 16). The percentage of proliferative cells in the entire crypt was similar in all three patient groups [269]. However, the labeling frequency in the upper two-fifths of the glands, was significantly higher in patients with ZES or CAG compared to controls (*p* < 0.01). 

The limited clinical data available and the colonic proliferative rate data from the studies reviewed above appear to conflict, with the former suggesting no increase in adenomas or CRC in ZES patients, but the latter showing increased mucosal proliferative rates. At present, with the limited data available, this discrepancy cannot be resolved without additional systematic clinical and laboratory studies of more ZES patients. This is not an inconsequential management question, because the answer will help address whether all ZES patients should undergo more careful colonic screening than is currently recommended if it is determined that they are at increased risk for colonic neoplasms.

#### 6.2.4. Other Effects of Chronic Hypergastrinemia in ZES: Gastrin and Pancreatic Cancer (Table 1)

Numerous findings from in vitro studies on various pancreatic cancer cell lines, in vivo animal studies, and studies of human pancreatic cancers, support the conclusion that gastrin stimulation may play an important role in human pancreatic ductal adenocarcinoma growth (PDAC), invasion, and pathogenesis [78,82,90,221,255,270,271,272,273]. Most PDACs ectopically overexpress CCK2R (gastrin receptors), and many also express CCK1R (CCK receptors), which gastrin has a low affinity for [78,82,221]. Gastrin has been shown to stimulate the growth of PDAC cells [78,82,90,221] in an autocrine manner [81,82,274]; and inhibition of gastrin by various methods including receptor antagonists, gastrin anti-sense, administration of gastrin- neutralizing antibodies, or gastrin receptor downregulation [82,271,273,275,276,277]. In man, whether an increased occurrence of PDAC occurs in chronic hypergastrinemic states is controversial, with various studies reporting an increased occurrence in some reports, while others report no association, from studies of patients with CAG/PA or patients taking long-term PPIs [278,279,280,281,282]. 

There are little data available to assess the possible frequency of occurrence of PDAC in ZES patients. In one NIH long-term study of 170 patients with sporadic ZES followed for a mean of 14 years, no patient developed PDAC [120]. In a recent surgical study of 52 ZES patients who were not cured post resection and underwent re-operation with mean follow-ups of 24 years from onset of ZES, two patients (4%) developed PDAC, which resulted in their deaths [107]. In the literature, only one case reports a patient with a history of ZES developed a PDAC [107,283]. Furthermore, in an analysis of survival/causes of death [42] in 106 MEN1/ZES patients and 1613 cases from the literature, no deaths for patients with PDAC were reported. Lastly, in the analysis of causes of death in 758 MEN1 patients [240] of which 28% had ZES, no patient was reported to have had PDAC. These limited data, in general, support the conclusion that ZES is rarely associated with PDAC. However, the recent reporting of PDAC in two ZES patient with active disease for over 24 years, raises the possibility that an association with very long-term follow-up may be found. Now that effective anti-secretory drug therapy can control acid hypersecretion in all ZES patients, thus patients are no longer dying from acid hypersecretory problems [28,118,120,284] and living longer, it will be important to continue to examine this association long-term. 

### 6.3. ZES as Model for Long-Term PPI Effects (Safety, Side-Effects, Effectiveness)

#### 6.3.1. Why is There a Need for Assessment of Long PPI Effects Particularly Related to Safety and Side-Effects?

As discussed in a previous paragraph, there is increasing debate of various safety and side-effect issues of PPIs in the last few years. In addition to the possible effects of chronic hypergastrinemia induced by the chronic PPI usage resulting in possible tumor growth in a number of different tissues, which was discussed above, a number of other safety/side-effect issues are receiving increased attention. A number of primarily observational/epidemiological recent studies report an association of chronic PPI use with the development of acute interstitial nephritis and chronic renal diseases, drug interactions and/or drug metabolism, and dementia [1,2,3,7,8,13,14,16,18], side-effects that appear not related to the chronic hypergastrinemia or to the acid inhibition [2]. Furthermore, a number of side-effects that are thought related to chronic acid inhibition with chronic PPI use are increasingly reported including: Pneumonia, increased gastrointestinal infections, bacterial overgrowth and changes in the gut microbiome, hypomagnesemia, decreased absorption of nutrients (vitamin B_12_, calcium, iron), gastric fundic gland polyps, development of rebound gastric acid hypersecretion when the PPI is stopped, spontaneous bacterial peritonitis, hepatic encephalopathy, and drug interactions in the gastrointestinal tract [1,2,3,7,8,13,14,16,18]. Other side-effects that are receiving increased attention due to chronic PPI use which are of unclear mechanism include an increased incidence of bone fractures [2,3,8,13,16,18,88].

#### 6.3.2. Why Could Results from the Long-Term Study of Zollinger–Ellison Syndrome (ZES) Provide Useful Insights into the Issues of Long-Term PPI Effects (Safety, Side-Effects, Effectiveness) in nonZES Patients?

First, ZES is an excellent model to study the long-term effects including efficacy, safety, and side-effects due to the chronic use of PPIs because >98% of these patients are now being treated with daily doses of PPIs [19,23,28,31,285]. PPIs are now the drugs of choice for treating these patients [28,122,123,124]. This has occurred because of their potency and long duration of action allows the gastric acid hypersecretion, in the majority (>98%) of ZES patients, to be controlled with once or twice a day dosing [28,30,31,33,35,286]. This is in marked contrast to the use of histamine H_2_-receptor antagonists, which are also effective in ZES patients, but which usually require high, frequent (every 4–6 h) dosing to be effective [28,52,53,187,287] (Table 1).

Second, ZES is a particularly useful model to assess the effect of very long/lifetime PPI treatment, because <30% of patients are cured lifelong [23,51,92,94,95], and in patients with continued tumor, the antitumor treatments do not result in complete disappearance of the gastrinoma [114,128,135,288]; thus, these patients have life-long hypergastrinemia with continuous gastric acid hypersecretion and require life-long anti-secretory treatment [28,92]. Life-long treatment occurs in every ZES patient treated with PPIs, because of its convenient dosing, and also because the use of histamine H_2_ receptor antagonists require titration of the drug dose by assessing the acid secretory rate prior to the next drug dose [28,52,53,106,187,188], which is not only inconvenient, but now is only offered in very few centers. 

Third, ZES patients were one of the groups of patients initially treated with PPIs when PPIs first became available in the 1980s [33,34,56,289] and therefore they are one of the few groups of patients who have been treated for >30 years continuously who are available for such long-term studies. Although numerous recent studies show the effectiveness and safety of PPI [1,2,7,8,9], these results are from studies generally with <5 years of mean follow-up, hence they do not address the questions related to very long PPI treatment.

Fourth, patients with ZES have a mean basal acid output >4-fold higher than normal [29,149], gastroesophageal symptoms, or diarrhea develop in every patient [126] and therefore their acid hypersecretion can be difficult to control sufficiently to render the patient asymptomatic [28,35,147]. This is especially the case in patients with complicate ZES )with MEN1/ZES, post Bilroth 2 gastric acid-reducing surgery, or with moderate/severe GERD symptoms) [35,125,147,148,290,291]. Because of the constant acid hypersecretion, which requires continuous control, monitoring the efficacy of the PPI or other anti-secretory drug is not difficult.

Fifth, a number of features of the care of these patients have made it easier to have safety/side-effect data of these ZES patients. ZES patients have to be monitored regularly and therefore the collection of safety data did not require additional patient interaction outside of the regular scheduled care. Similarly, many of these patients undergo UGI endoscopy at regular intervals and therefore gastric mucosal changes are followed in many patients as part of their routine care schedules. 

#### 6.3.3. Chronic PPI Treatment and Nutrient Malabsorption (vitamin B12 (VB_12_), Iron, Calcium, Magnesium [Mg]) (Table 1)

##### Chronic PPI Treatment and Nutrient Malabsorption (VB_12_, Iron, Calcium, Mg): General

The absorption of VB_12_, iron calcium, and Mg are decreased in hypo-/achlorhydric conditions [18,292]. Specifically, gastric acid secretion is needed for dietary VB_12_ absorption from food due to its effect on the activity of pancreatic proteases to cleave the VB_12_ from protein [18,292]. Animal studies and some human studies show the gastric acid secretion can facilitate calcium absorption [18,292]. Also, gastric acid facilitates the absorption of non-heme iron in food [18]. Body Mg stores are determined by a balance between renal excretion and intestinal absorption [292]. Several studies report that chronic PPI treatment can increase both urine and fecal Mg losses, however the exact mechanism is not clear at this point [292]. 

The effects of potent acid anti-suppressants such as PPIs in man, long-term on each of these nutrients is controversial. This confusion exists because with VB_12_, iron, and calcium, some studies, but not others, show decreased absorption/body stores with chronic PPI use [14,18,292,293,294]. There are numerous reports of hypomagnesemia with chronic PPI treatment, however the exact mechanism, or why it occurs in some patients, and not in others, is not clear [14,18,292,293,294]. 

##### Chronic PPI Treatment and Nutrient Malabsorption: VB_12_ (Table 1)

**Figure 4:**
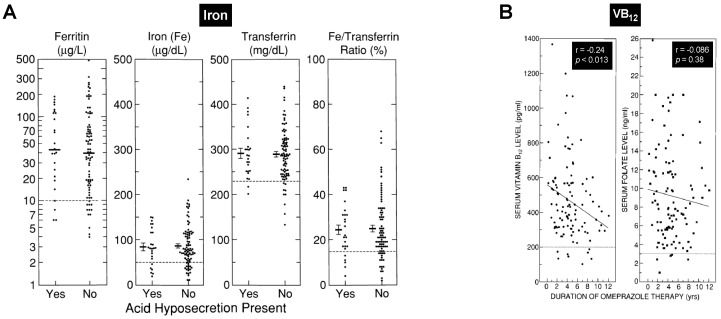
Effect of prolonged PPI treatment in ZES patients on body iron stores/levels (**A**) or serum vitamin B_12_ and folate levels in two NIH (**B**). In (**A**), data from 109 ZES patients treated with antisecretory drugs for a mean of 10.2 years are shown (81% PPIs for 6 years, 8% histamine H_2_ receptor antagonist, 10% cured, off all drugs). Results are shown for patients with or without advanced basal acid hyposecretion on the antisecretory drug. Normal values are shown by the dotted lines and means ± SEM are also shown for each group. (**B**) shows serum vitamin B_12_ and folate levels in 108 ZES patients correlated with time of omeprazole treatment. The serum vitamin B_12_ levels inversely correlated with the time of omeprazole treatment, whereas there was no effect on the serum folate levels. Figures are drawn from data in [27,253]. These data showed that prolonged chronic treatment in ZES patients did not affect serum iron levels or iron body stores, however there was a significant decrease in serum vitamin B_12_ levels correlating with PPI treatment duration, but not serum folate levels.

Despite the life-long treatment of ZES patients with PPIs, there are only a few studies that have evaluated their effects on body stores/absorption of these nutrients in these patients. In the case of VB_12,_ one NIH prospective study of consecutive ZES cases [27] reported results after a mean of 4.5 years of PPI treatment or up to 10 years of histamine H_2_ receptor antagonist treatment in 131 ZES patients. In this study [27], there was no alterations in serum folate or hematological parameters, however VB_12_ levels were significantly reduced in PPI treated patients compared to those taking histamine H_2_ receptor antagonists (*p* = 0.03) (Figure 4, right panels). The degree of the decrease in VB_12_ levels showed an inverse relationship to the degree of acid hyposecretion with patients treated with PPIs showing sustained hyposecretion having lower levels VB_12_ levels (*p* = 0.0014) and the lowest levels in patients treated with PPIs with complete achlorhydria (*p* < 0.0001). In a subgroup of 68 patients with two VB_12_ determination at least five years apart, the serum VB_12_ levels decreased by 68% [27]. Furthermore, the duration of PPI treatment correlated inversely with the serum VB_12_ levels, but not the serum folate levels [27] (Figure 4, right panels). In a retrospective study [295] of 61 patients with hypersecretory states (45 = ZES, 15 = other) who had chronic treatment (31% with a mean of eight years), 10% were found to have had low VB_12_ levels and 31% had normal VB_12_ levels with VB_12_ deficiency)(decreased serum homocysteine levels with normal folate levels). No correlation was seen between changes in serum VB_12_ levels and duration of PPI treatment in this study or the degree of acid suppression [295]. Therefore, while these two studies on ZES patients treated long-term with PPIs show an increased rate of VB_12_ deficiency, they come to different conclusions on the mechanism. The NIH study [27] provides evidence that it is directly related to the PPI treatment through duration of treatment and degree of acid suppression, whereas the other study [295] does not support this conclusion that support that the VB_12_ deficiency due to acid-related, but does not establish the mechanism of the deficiency.

The divergence of results in the two ZES studies of VB_12_ deficiency with chronic PPI treatment mirror the difference in results of the possible effects of chronic PPI treatment in VB_12_ level/stores in non-ZES patients [14,18,292,293,294]. In these studies, many [14,18,296,297] but not all [13,18,298,299] report a 2–4-fold increased risk of VB_12_ deficiency/low serum VB_12_ levels with chronic PPI treatment [13,294]. An important factor that is not considered in most studies in the US is the widespread use of multivitamin tables is not assessed and this can have an impact on the results as it will counter a decrease in absorptive changes because crystalline VB_12_ in these tablets is not affected by the acid secretory status. 

##### Chronic PPI Treatment and Nutrient Malabsorption: Iron (Table 1)

There are only limited data on the effects of chronic PPI treatment on body iron levels in ZES patients. One needs to remember that ZES patients have multiple other reasons that they can altered body iron stores including; that the majority present with abdominal pain due to either GERD or peptic ulcer disease [116,126], each of which can result in chronic or acute blood loss; the gastric acid hypersecretion can result in malabsorption of nutrients [21,126,300]; the chronic uncontrolled acid hypersecretory state can result in a general effect on wellbeing including appetite [116]; on occasion the tumor can result in blood loss [301]*;* and lastly, a significant proportion of these patient are taking multivitamin tables with various minerals including iron, which can affect body stores and mask a decrease in absorptive changes. In an NIH study [302], body iron stores (serum iron, ferritin, transferrin, iron/transferrin ratio, complete blood counts) were assessed in 109 consecutive ZES patients without any previous gastric resections who were being treated long-term with either PPIs (78%), histamine H_2_ receptor antagonists (10%), or receiving no acid anti-secretory treatments because there were post curative gastrinoma resection (10%). These patients had a mean duration of 5.7 years of PPI treatment and a total duration of any anti-secretory drug treatment of 10 years [302]. Acid hyposecretion post anti-secretory drug(<0.2 mEq/hr output for hour prior to next drug dose) was present in 45% of the patients, however there were no differences in any of the iron parameters or blood counts between patients with or without acid hyposecretion, taking or not taking PPIs, or with different drug duration treatments (Figure 4, left panels). The study concluded that PPI treatment for up to six years or anti-secretory drug treatment of any kind for 10 years, does not cause decreased body iron stores or iron deficiency in ZES patients [302]. This result was in marked contrast with the findings in another similar study [27] where with ZES patients with similar characteristics, similar degrees of acid hyposecretion after PPIs or other anti-secretory drug treatment, similar PPI dosage and duration of PPI treatment showed a marked decrease in VB_12_ levels [27]. There are no other studies on iron body stores in ZES. 

The lack of effect of PPIs on iron stores of patients with ZES found in the NIH study [302] and discussed in the previous paragraph, agrees with some recent studies in non-ZES patients [299,303], however it disagrees with number of recent studies reporting chronic PPIs effect on iron body stores in a number of disease states. Chronic PPIs were associated with a reduction in absorption of non-heme iron in patients with hereditary hemochromatosis [304] who are treated with phlebotomies because they have increased intestinal iron absorption. PPI treatment led to a decreased need for phlebotomies [304]. Similarly, patients with congenital dyserythropoietic anemia have ineffective erythropoiesis and have increased iron absorption leading to iron overload, which may require treatment by iron-chelating therapy [305]. Treatment of eight such patients [305] with PPIs decreased serum ferritin and reduced mean hemoglobin levels led the authors to conclude it was reducing iron overabsorption and my lower the need for chelation therapy. A third study reported the effect of chronic PPI treatment of patients after sleeve gastrectomies, who frequently develop iron deficiency anemia post-surgery [306]. The PPI-treated patients (*n* = 85) compared to a group not taking PPIs (*n* = 118) had a 3.3-fold higher incidence of iron deficiency anemia [306]. In a fourth study [307], a retrospective study of patients with iron deficiency anemia taking PPIs (*n* = 50) was performed in which they assessed the hematological response to ferrous sulfate treatment. Only 16% of patients treated with PPIs had a normal rise in hemoglobin levels and 40% a normal response to increases in ferritin level with iron treatment [307], leading the authors to conclude the PPIs were decreasing the absorption of oral iron resulting in decreased erythropoiesis [307]. Lastly, the effect of chronic treatment with PPIs was assessed in renal transplant patients (*n* = 646) who have a frequent occurrence of iron deficiency anemia [308]. The PPI-treated group (*n* = 363) had lower serum iron levels (*p* < 001), lower serum ferritin levels (*p* < 0.001), and lower hemoglobin levels (*p* = 0.007). Multivariate analysis showed that PPI use was independently associated with the development of iron deficiency anemia (OR—1.6) [308]. Furthermore, the PPI effect was dose-related with a greater occurrence of iron deficiency anemia (OR—2.3) with a high PPI dose than a low PPI dose (OR—1.8). The authors concluded that this effect is due to the PPI’s impairing iron absorption [308] 

A recent large community-based case-control study compared acid anti-secretory drug use in 77,046 subjects with iron deficiency anemia to 389,3140 subjects without iron deficiency anemia [309]. The use of either PPIs or a histamine H_2_ receptor antagonist for ≥2 years was associated with an increased risk of iron deficiency anemia and the risk increased with increasing potency of acid inhibition and decreased after the anti-secretory drugs were stopped [296]. Another case-control study investigated the risk of iron deficiency anemia with the use of PPIs in a UK clinical Practice database [310]. In this comparison, there were 2960 chronic PPI users who had taken the PPI continuously for at least one year, a PPI limited group (*n* = 6607) who intermittently used PPIs, and a PPI nonuser group (*n* = 20,657). The rate of occurrence of iron deficiency anemia was 3.9-fold higher in the continuous PPI users and 1.71 -fold higher in the intermittent PPI users compared to the non-PPI users, thus showing a dose response affect. 

These latter studies support the conclusion that PPIs can have a clinical effect on iron absorption in man. At present, it is unclear why this was missed in the ZES patients in the NIH study, but because of the numerous aspects of this disease that could affect body iron status in these patients, ZES may not be a good model for assessing chronic PPI treatment’s effect on body iron stores and iron absorption. 

##### Chronic PPI Treatment and Nutrient Malabsorption: Mg (Table 1)

Numerous studies, case reports, and two different meta-analysis report an association of chronic PPI use with the development of hypomagnesemia, which in rare cases can be severe and life-threatening [13,14,18,292,293,294,311,312,313]. The exact frequency of its occurrence is not clear, but in various studies it has been estimated as occurring with an increased frequency with a pooled risk ratio (RR) of 1.8 [311], a second systematic review calculated a RR of 1.44 in patients with chronic use of PPIs [312], and a final study also reported a 43% increase in the incidence of hypomagnesemia in patients taking PPIs over matched controls [314]. In one analysis [315], it comprised 1% of the 66,102 adverse PPI events reported to the FDA. At present, it is not clear which patients will develop hypomagnesaemia with chronic PPI use, although it has been reported to be more frequent in patients concurrently taking diuretics [18,313,314,316], a greater occurrence in males, longer duration of taking the PPI, and in the elderly [292,315]. Its strong association with PPI use is supported by the observation that when the PPI is stopped, the hypomagnesemia disappears [18,313,317]. Evidence suggests the development of the hypomagnesemia is a general class effect, because it was reported with all PPIs except esomeprazole [315] and it recurred with substitution of another PPI, but not when a histamine H_2_-receptor antagonists is used [18,313,318]. The exact mechanism of the hypomagnesemia is not clear [18,292,319,320], with studies generally showing no increased renal secretion, while other studies have suggested/provided evidence for increased GI losses of magnesium with chronic PPI use [18,292,319,320,321]. It has recently been shown that the regulation of transient receptor potential melastatin-6 transporters (TRPM) in the colon by PPIs may explain their effects on Mg homeostasis [319,320,322]. Mg active transport in the GI tract is regulated by TRPM cation channels 6 and 7 and they are up regulated by activation of colonic H^+^K^+^ATPase, and it is proposed that PPIs inhibition of colonic H^+^K^+^ATPase results in decreased TRPM activity, causing hypomagnesemia in some patients [319,320,322]. Recent animal studies provide evidence that PPI-induced changes in the gut microbiome combined with a low dietary Mg intake are also important in the pathogenesis of PPI-induced hypomagnesemia [323].

There have been no systematic studies of hypomagnesemia in ZES patients. There are three case reports of patents with ZES developing hypomagnesemia with chronic PPI treatment [324,325]. The three patients reported had PPI-induced hypomagnesemic features consistent with those described and reviewed above in non-ZES patients. Specifically, the hypomagnesemia resolved when the PPI was replaced by a histamine H_2_-receptor antagonist; recurrence of the hypomagnesemia was seen if the PPI was restarted; and the hypomagnesemia caused severe symptoms in some patients [324,325]. In the prospective NIH ZES studies, which have involved more than 250 ZES patients treated long-term with PPIs, only one case (not reported) of hypomagnesemia thought due to PPI treatment was seen, which results in a rate of 0.4% of patients treated. This suggests that the rate of developing hypomagnesemia in these patients is low even though many take higher doses of PPIs (40–100 mg/day-omeprazole equivalent dose) [30,35,189], 25% take the dosing BID [30,35,189], and the mean treatment time is >10 years. These results suggest this side-effect is not clearly dose-related or duration-related in these patients. The above rate PPI-induced hypomagnesemia in the NIH ZES patients is considerably lower than the rate of 15–55% reported in some series of non-ZES patients [314].

##### Chronic PPI Treatment and Nutrient Malabsorption: Calcium (Table 1)

Gastric acid secretion resulting in an acidic pH has been shown in numerous studies to markedly increase the dissolution and ionization of poorly soluble calcium salts and when absent, decreased ionization and calcium absorption can occur, resulting in osteoporosis with decreased bone density [18,326,327]. The effect of acid is important because only ionized calcium is absorbed in the small intestine [327]. In various human conditions resulting in hypo-/achlorhydria, such as CAG, pernicious anemia, post-vagotomy or post partial gastrectomy, and increased occurrence of osteoporosis as well as bone fractures, is seen [18,327,328]. Numerous studies in animals support the conclusion that potent gastric acid suppressant drugs such as the PPIs, can also result in decreased calcium absorption [18,327,329]. However, studies in humans treated with PPIs and other acid anti-secretory drugs have yielded conflicting results with some studies showing these drugs decreased calcium absorption and others that they had no effect on calcium absorption [18,330,331,332]. The effect of PPIs on calcium absorption and metabolism is particularly controversial and an important subject because numerous studies [18,328,330,333], but not all [330,334,335,336,337,338], have reported that patients treated with PPIs have an increased occurrence of bone fractures, particularly of the vertebral column and hip. Three recent meta-analysis [339,340,341] all show that fractures of the hip or spine are more frequent with chronic PPI use with a risk ratio (RR) for hip fractures of RR = 1.2 [339] and in two other studies RR = 1.26, *p* < 0.0001 [340,342], and RR = 1.58 (for spine fractures) [340]. A recent population [341] matched cohort study involving 10,596 patients demonstrated an increased risk of osteoporosis, hip, or vertebral fracture with stroke. The adjusted hazard ratio (HR) for osteoporosis was HR = 1.25 (*p* < 0.01), hip fracture HR = 1.18 (*p* = 0.048), and vertebral fracture (HR = 1.33; *p* < 0.001) [341]. For each of these three outcomes, the highest PPI dose was associated with the highest risk [341]. At present, there is no agreement of the mechanism by which PPIs could be causing the increase occurrence of bone fractures [18,292,330,337]. Proposed mechanisms include chronic PPI use leading to decreased intestinal absorption of calcium resulting in an increase in bone fracture rate; PPI-induced inhibitory effects on the vacuolar type of H^+^K^+^ATPase of osteoclasts leading to an increase in their activity; PPI-induced hypochlorhydria leading to hypergastrinemia, which in turn stimulates the development of parathyroid hyperplasia; PP-induced hypomagnesemia, which can contribute to altered bone metabolism and increased risk of bone fractures; and the hypergastrinemic stimulation of ECL hyperplasia resulting in an increased in histamine production, which can stimulate proliferation of osteoclasts resulting in increased bone absorption [18,292,293,294,330]. More recently, it has been proposed that low VB_12_ levels, which can be associated with PPI treatment [297], affects skeletal fragility through the modulation of collagen cross-linking independently of areal bone mineral density [343]. This increased bone fragility coupled with recently evidence reporting an increased risk of falling in elderly patients with PPIs use may be contributing to increasing bone fracture rates [343,344,345,346]. Although a placebo controlled, double-blind trial in postmenopausal females found decreased calcium absorption with PPI treatment, the results suggesting PPI causes osteoporosis are largely negative [18,334,347,348], as are studies reporting PPI treatment does not cause changes in bone mineral density or bone structure that would predispose to fracture [348].

There are no studies in ZES patients on fractures related to chronic PPI use. In general, ZES patients will not be a good model to study this association, even though they have life-time treatment with PPIs, frequently with higher PPI doses, for a number of reasons, which will overall confound the determination of present/cause of bone changes/osteoporosis or bone fracture. First, the delay in diagnosis for ZES patients is 6–7 years [29,116] and during this time, diarrhea frequently develops and may be associated with malabsorption [21,29,149,300,349]. This malabsorption can affect mineral/calcium absorption also so that these patients may develop an additional cause independent of any anti-secretory drug for bone changes/fractures [350]. Second, in the 25% of patients who have MEN1/ZES, hyperparathyroidism is present in 95–100% [116,145] and this can cause widespread bone changes, including the development of osteoporosis leading to increased occurrence of fractures [351,352,353,354]. Third, these patients have an increased occurrence of a number of features that have been proposed as mediating the PPI induced fractures [18,292,293,294,330]. These effects include the fact that all ZES patients have gastric ECL cell proliferative changes, often of advanced grade [25,201], which could be a source of increased histamine release increased in histamine production, which can stimulate proliferation of osteoclasts resulting in increased bone absorption [18,292,293,294,330]. Also, vitamin B_12_ deficiency is not infrequent in ZES patients [27], which also has been proposed as a mechanism for the PPI induced bone effects by affect bone fragility as discussed above. Therefore, the presence of ZES introduces additional causes for effects on calcium homeostasis and/or bone metabolism, which could contribute to bone fractures and thus confounds the ability to determine the possible role of PPIs only in these processes. 

#### 6.3.4. Chronic PPI Treatment and Other Reported PPI Side-Effects and Insights from Studies of ZES Patients (Table 1)

Recently, in addition to the above discussed side-effects of chronic PPI treatment, a number of other prominent side-effects are also receiving considerable attention. These include: An increased occurrence of GI infections particularly with *Clostridium difficile* and the development of small intestinal bacterial overgrowth; spontaneous bacterial peritonitis; nontyphoid salmonella and campylobacter infections; development of pneumonia; the development of acute and chronic renal diseases; the development of dementia; effects on drug metabolism that have clinical significance; an increased risk of cardiovascular problems including myocardial infarction, stroke, and cardiovascular death; and rebound acid hypersecretion [14,18,292,293,294]. As pointed out above, most of these associations are based on observation/epidemiological studies not proving causality and a number of recent studies and consensus papers have provided evidenced from chronic PPI studies of 3 to <10 years of the safety of PPIs and concluded in most cases no additional routine testing is necessary with PPI treatment up to these times [8,9,14,292,294].

There are no specific studies on any of the areas in ZES patients and it is not apparent that in most cases the study of ZES patients will offer any advantages. One area where some insight may be provided is the question of rebound acid hypersecretion when PPIs are stopped after chronic long-term use. The possibility of rebound hypersecretion is receiving increased attention because it may be one of the mechanisms contributing to the difficult of stopping PPIs in patients. A number of studies, but not all, have reported with chronic PPI use in non-ZES patients that when the PPI is stopped, there can occur increased symptoms of acid hypersecretion (GERD, etc.) and the presence of acid rebound hypersecretion can be found [19,143,355,356,357,358,359,360]. The occurrence and significance of rebound hypersecretion post PPI chronic treatment is controversial because different studies report conflicting results [19,143,355,356,357,358,359,360]. Also, most of the studies are of short duration (<3–5 years) and therefore, whether this will become an increasing problem with very long-term, lifetime PPI treatment is not clear. Studies from ZES patients that are cured (sporadic ZES) post-surgical resection of the gastrinoma) from NIH may be one model, because of their very long-term hypergastrinemia prior to cure, that may provide insights pertain to the above areas of uncertainty. Two NIH studies in sporadic ZES patient provide some insights into this subject. One study [144] examined acid secretory changes in 20 sporadic ZES post curative resection [92,95,130,132] (normal fasting serum gastrin [24], negative secretin provocative test [361], no imageable tumor). These patients had a mean preoperative BAO of 39 mE/hr and MAO of 56 mEq/hr, which decreased by 75% and 50%, respectively, at 3–6 months post curative resection, and remained unchanged for four years of follow-up [144]. However, at four years of follow-up, the BAO had not returned to normal in 67% of the patients and they remained acid hypersecretors, even though there was no evidence of residual gastrinoma [144]. A second NIH study [143] attempted to examine mechanisms for this continued acid hypersecretion in a large percent of cured sporadic ZES patients. In that study [143], 50 ZES patients who had a mean disease duration of 14 years prior to surgery were studied after a minimum follow-up of two years after a post curative resection (mean postoperative follow-up of four years). At the last follow-up, 38% of the cured patients had become basal acid normosecretors, with 62% showing some degree of continued basal acid hypersecretion, including 28% of patients having residual extreme hypersecretion (≥25 mEq/hr) [143]. The postoperative hypersecretory group had greater continuing postoperative ECL proliferative changes, higher serum chromogranin A levels, and higher 24-hr urinary N-MIAA (histamine breakdown product) secretion. It was concluded that prolonged chronic hypergastrinemia in man can lead to changes in ECL cells that are either irreversible or sustained by some unknown mechanism [143]. These data raise the possibility that a similar phenomenon may be seen in non-ZES patients after very long duration of PPI treatment (>10 years). 

## 7. Conclusions

The long-term studies of patients with ZES support the proposal that they are a good model to study the long-term effects of chronic hypergastrinemia and chronic PPI use in man. Greater than 70% of these patients are not cured even with increasingly sensitive tumor imaging modalities, their diagnosis continues to be delayed 5–7 years, and is getting more difficult and delayed because of the widespread use of PPIs and the control of the acid hypersecretion in all patients has resulted in overall mean survivals >30 years from diagnosis with the result these patients allow very long follow-up. During this time, >95% are treated with chronic daily administration of PPIs so they not only allow long-term data on chronic hypergastrinemia, but also the efficacy/side-effects of PPIs. Study of these patients provides insights in a number of important areas related to concerns about the long-term use of PPIs especially in non-ZES patients with moderate/advanced GERD who are increasingly being treated with PPIs for >5–10 years. Studies of the ZES patients have provided insights into the long-term effects of hypergastrinemia on the gastric mucosa and the possible risks of gastrin-enhanced growth effects particularly on stimulating the development of gastric neuroendocrine tumors, as well as other tumors and also on the nutritional effects of chronic hypo-/achlorhydria, rebound acid hypersecretion, and the occurrence of a number of other proposed PPI-related side-effects. In general, these studies are consistent with the recent reports of the continued efficacy and safety of PPIs in non-ZES patients from studies usually <5 years mean treatment. However, both studies in animals as well as some findings from chronic hypergastrinemic states in man including from ZES patients, support proposals that only very long-term careful prospective studies will fully resolve all of the issues leading to the controversies currently being raised about lifelong PPI use.

## Figures and Tables

**Table 1:** Summary of potential side-effects of PPIs and insights from studies of patients with gastrinomas causing ZES with chronic hypergastrinemia (Chr. HG) and with acid hypersecretion controlled by very long-term treatment with PPIs.

Potential PPI Side-Effect	Potential Mechanism	Insights from ZES Studies
**General** **Why ZES useful model of Chr HG**	Chronic hypergastrinemia (Chr. HG)	ZES patients have lifelong Chr. HG Delayed diagnosis-6yrs Less 20% cured lifelong 30–40% = FSG levels in range of PPIs in non-ZES pts, rest have > FSG levels All forms of circulating gastrin including amidated, NH2,COOH extended forms
**General** **Why ZES useful model of long-term PPI use**	Lifelong need for potent gastric antisecretory drugs—PPIs drug of choice	>80% ZES patients take PPI lifelong Frequently take hi doses PPI Regularly followed for acid control and other side-effects
**ECL hyperplasia/gastric carcinoids**	Chronic hypergastrinemia (Chr. HG)	All ZES patients have ECL hyperplasia Advanced ECL changes including dysplasia is seen both sporadic and MEN1/ZES ECL changes are more advanced in MEN1/ZES Carcinoids are very uncommon in sporadic ZES which resemble non-ZES PPI users 23% of MEN1/ZES have gastric carcinoids Sporadic ZES more resembles chronic PPI users than CAG/PA patients which have high incidence of inflammation ± atrophy and frequently develop carcinoids
**Esophageal, gastric, pancreatic adenocarcinomas**	Chronic hypergastrinemia (Chr. HG)	Limited data but no evidence for increased incidence in ZES
**Colorectal cancer**	Chronic hypergastrinemia (Chr. HG)	Limited data but no evidence for increased incidence in ZES2 Two studies show increased rectal/colonic mucosal increased proliferative rates in ZES
**Nutrient malabsorption (Fe, Ca)**	PPI-induced Hypo-/Achlorhydria	Limited data but no evidence for malabsorption in ZES
**Nutrient malabsorption (VB12)**	PPI-induced Hypo-/Achlorhydria	Two studies report decreased VB12 levels in in ZES2 One ZES study correlates low serum levels in VB12, but not folate levels with use of PPIs, duration of PPI use and with the degree of PPI-induced acid hyposecretion.
**Hypomagnesemia**	Unclear = mechanism	Uncommonly reported in ZES patients showing that it is not dose-related or related to length of treatment with PPIs
**Bone fractures**	Unclear = mechanism	No data in ZES2 ZES poor model to study this in as 20–25% have MEN1 with hyperparathyroidism, which causes bone disease; a proportion have malabsorption prior to diagnosis which could contribute to bone disease, and high proportion had low VB12 levels from long-term PPI that could contribute
**Rebound hypersecretion after stopping PPI**	Unclear = mechanism	Studies of ZES patient’s post-curative resection of gastrinoma have provided insights into very long-term effects of PPI treatment/Chr HG on post drug acid secretory effects.

Abbreviations: Chr HG—chronic hypergastrinemia, ZES—Zollinger–Ellison syndrome, PPI—proton pump inhibitor, ECL—gastric enterochomaffin-like cell, MEN1/ZES—Zollinger–Ellison syndrome in patients with Multiple Endocrine Neoplasia-Type 1, VB12—vitamin B12, Fe—Iron, Ca—calcium, Mg—magnesium, pts—patients.

## Abbreviations

BE	Barrett’s esophagus
CCK2R	Cholecystokinin type 2 receptor (gastrin receptor)
CgA	Chromogranin A
CGA/PA	Chronic atrophic gastritis/pernicious anemia
CRC	Colorectal cancer
EAC	Esophageal adenocarcinoma
ECL cell	Gastric enterochromaffin-like cells
FSG	Fasting gastrin concentration
GC	Gastric cancer
GER	Gastroesophageal reflux disease
Gly-gastrin	Glycine-extended gastrin
HP	Helicobacter pylori
Mg	Magnesium
MEN1/ZES	Multiple Endocrine Neoplasia type 1 with ZES
PDAC	Pancreatic ductal adenocarcinoma
PPI	Proton pump inhibitor
VB_12_	Vitamin B_12_
ZES	Zollinger–Ellison syndrome
